# Reimagining Pt(II) Anticancer Agents: The Role of Ferrocene in Monofunctional Chemotherapeutic Compounds

**DOI:** 10.1021/acs.inorgchem.5c00704

**Published:** 2025-06-02

**Authors:** Selma Miguel, Javier Ortín-Fernández, Silvia Gómez-Pastor, María Ángeles Moliné, Pedro A. Sánchez-Murcia, Inés Corral, Francisco Sanz-Rodríguez, Ana María González-Vadillo

**Affiliations:** † Departamento de Química, Facultad de Ciencias, Universidad Autónoma de Madrid, Madrid 28049, Spain; ‡ Departamento de Química Inorgánica, Facultad de Ciencias, Universidad Autónoma de Madrid, Madrid 28049, Spain; § Departamento de Biología, Facultad de Ciencias, Universidad Autónoma de Madrid, Madrid 28049, Spain; ∥ Laboratory for Computer-Aided Molecular Design, Division of Medicinal Chemistry, Otto Loewi Research Center, Medical University Graz, Neue Stiftingtalstrasse 6/III, Graz 8010, Austria; ⊥ BioTechMed-Graz, Mozartgasse 12/II, Graz 8010, Austria; # Institute for Advanced Research in Chemical Sciences (IAdChem), Universidad Autónoma de Madrid, Madrid 28049, Spain

## Abstract

The side effects and resistance to treatments associated with platinum compounds, such as cisplatin, underscore the present need for novel anticancer agents with improved properties. The development of hybrid drugs, combining two bioactive units, offers a promising approach by synergistically enhancing the biological activity of the two fragments while reducing the resistance of classic drugs. This work presents the synthesis of a novel family of heterobimetallic compounds, featuring a monofunctional Pt­(II) complex with amino groups and a *p*-ferrocenylaniline ligand. Cytotoxic assays reveal that the derivatives with methylated and isopropylated substituted amines exhibit remarkably higher activity against several tumor cell lines compared with cisplatin and the unsubstituted diamino complex. Notably, these methylated and isopropylated complexes demonstrate high selectivity and present high antitumor activity in cell lines where cisplatin is ineffective. Classical molecular dynamics simulations targeting DNA reveal a consistent relation between the extent of distortion of the duplex upon complex coordination and the cytotoxic activity observed in biological assays. According to our simulations, coordination of the heterometallic complexes can produce a significant disruption of the H-bond pattern of the platinated guanine. Moreover, the distortion mechanism induced by the voluminous substituents of the amino ligands entails either the intercalation of the ferrocene moiety, facilitating new hydrogen bonds between originally non-interacting base pairs and new weak attractive stacking interactions between the Pt­(II) complex and neighboring nucleobases, or the displacement of adjacent nucleotides from the pairing region toward the solvent environment.

## Graphical abstract

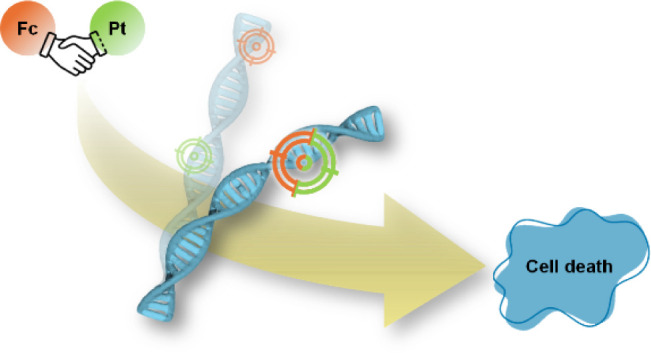

## Introduction

After ischemic heart disease, cancer is the second leading cause of mortality in the world today. According to the World Health Organization, cancer was responsible for one out of six deaths in 2020, with breast, colon, lung, rectum, and prostate cancers at the forefront.^1^ Despite the existence of various strategies, the use of chemotherapy, either standalone or in a multimodal version, combined with other therapies, prevails as one of the most powerful approaches to treat cancer.^2^

Chemotherapy employs organic compounds or/and metallodrugs to destroy cancer cells or inhibit their proliferation. The main mode of action of metallodrugs involves their covalent binding to DNA after activation by aquation.^3,4^ DNA distortion induced by drug attachment induces the arrest of the cell cycle, activating repair mechanisms or provoking cell death via apoptosis.^4^ Furthermore, protein platination might also contribute to the pharmacological effect of these drugs.^5^

The worldwide approved cisplatin and its analogs carboplatin and oxaliplatin (Figure 1) are included among the most active and effective Pt-based drugs for the treatment of solid tumors.^6^ Other cisplatin analogs, as nedaplatin, lobaplatin, and heptaplatin, have been only approved in Japan, China, and South Korea, respectively.^7^ All of these complexes are known to cause bifunctional lesions to DNA, by covalently linking two guanine nucleobases from the same or different strands. Unfortunately, all these Pt derivatives show important adverse side effects such as neurotoxicity, ototoxicity, and nephrotoxicity caused by the increase of singlet oxygen levels and neuron DNA damage. Additionally, prolonged treatments can lead to intrinsic and acquired resistance due to decreased cellular uptake or increased DNA repair. To overcome these drawbacks the search of new improved drugs is encouraged.^8–11^

**Figure 1:**
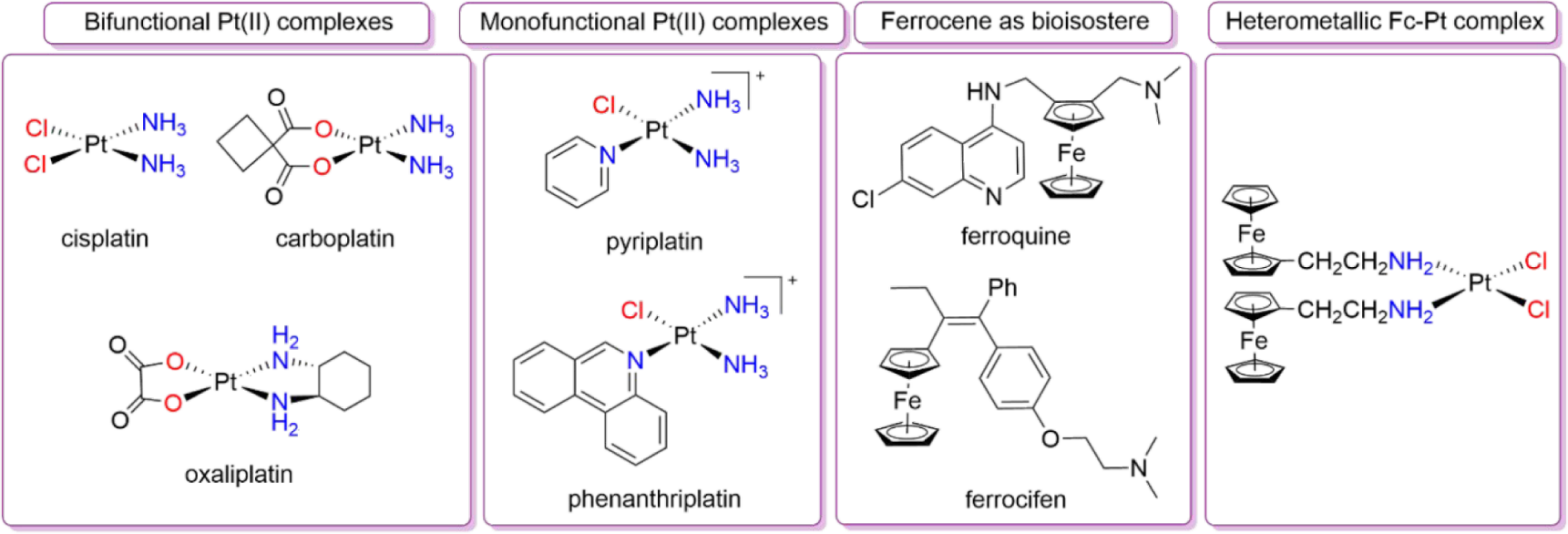
Selected platinum and ferrocene compounds with biological activity.

For these reasons, alternative complexes, containing other metals, such as ruthenium, gold, or palladium, have been considered.^12–15^ Nevertheless and despite the plethora of potential anticancer drugs proposed, up to now, no coordination compound has been able to overtake the effectiveness of platinum drugs.

Another significant avenue for the development of improved anticancer drugs considers exploring the pharmacological effects of structurally modified platinum complexes that already exist. Among them, monofunctional platinum (II) compounds, allowing a different mode of action due to their single coordination of DNA, are considered a promising alternative approach to overcoming the disadvantages of bifunctional platinum complexes. Pyriplatin and phenanthriplatin (Figure 1) were the first reported complexes of this kind that approached or even surpassed the anticancer activity of cisplatin. Studies have shown that these monofunctional complexes not only have DNA as their target but also they interact with other biomolecules such as topoisomerases and DNA and RNA polymerases.^16–19^ Along this vein, the design of novel multifunctional compounds highly biosafe and with multimodal therapeutic performance is currently a very active area of research.^20,21^

It should be also noted that, in recent decades, bioorganometallic chemistry has significantly boosted the development of new compounds with medical application by synergically combining the pharmacological properties of organometallic compounds and cisplatin-related coordination complexes.^22,23^ These novel binuclear complexes present a greater lipophilicity, facilitating cell incorporation processes, are able to change their oxidation state and can target biomolecules other than DNA. Among transition metals, iron, in the form of ferrocene, stands out as a key unit in this field.^24^

Owing to its extraordinary physicochemical properties, ferrocene serves as a privileged motif to synthesize compounds with a broad spectrum of applications.^25–27^ As a matter of fact, ferrocene derivatives have demonstrated pharmacological potential including, among others, antimicrobial, antimalarial, and antitumor activity.^26,28–31^ Two strategies have been successfully implemented in the design of ferrocene-based drugs with improved performance. Both rely on the ability of ferrocene to participate in redox processes, which contributes to their mechanism of action. The reversibility of the oxidation–reduction process of ferrocene can alter the cellular redox balance through reactive oxygen species (ROS) generation, prompting DNA oxidative damage. Additionally, ferrocene can disrupt the cell cycle, induce apoptosis, and inhibit thioredoxin reductase.^32,33^ The first of these two strategies, with ferroquine^34^ and ferrocifen^35,36^ as main representatives, integrates ferrocene as a bioisostere (Figure 1). In fact, the former is a structural analogue of chloroquine and, thus, exhibits antimalarial activity, while the second is a structural analogue of hydroxytamoxifen and active in both hormone-dependent and -independent breast cancer cells. The second strategy entails the development of hybrid molecules for multimodal targeting.^37^

Hybrid molecules consist of at least two bioactive units each one targeting different biological pathways.^38,39^ In the context of drug activity and more particularly in chemotherapy, metals play a central role. Consequently, the synergistic effect produced by the concurrence of several metals in the same compound can enhance the biological activity, increase the selectivity, or mitigate cell resistance compared to their mononuclear counterparts, by combining different mechanisms of cell death and/or interactions with several targets.

Given this context and with the long-term goal of investigating the potential therapeutic impact of modified platinum complexes, we aim to explore the antineoplastic effect of novel drugs combining Pt­(II) units and ferrocene. Several years ago we demonstrated that *cis*-[PtCl_2_{Fe­(η^5^-C_5_H_4_(CH_2_)_2_NH_2_)­(η^5^-C_5_H_5_)}_2_], which incorporates ferrocene amines as nonleaving ligands, exhibited superior cytotoxic activity compared to cisplatin against HeLa cervical and SW1573 alveolar cancer cell lines.^40^ Furthermore, this compound did display notable efficacy against the cisplatin-resistant WiDr colon cancer cell line. Of particular interest, these heterometallic platinum­(II) complexes were proven not to cause cell cycle arrest in the S-phase, as occurs with cisplatin, suggesting a different mode of action.^40^

With the aim of broadening the landscape of heterometallic platinum complexes and exploring their potential as antitumor agents, we have chosen to investigate the impact of incorporating *N*-aromatic linkers connecting the ferrocene framework and the platinum core. Herein, we describe the synthesis and characterization of this novel family of monofunctional platinum­(II) complexes carrying a *p*-ferrocenylaniline ligand. We also assessed the therapeutic potential of these complexes by undertaking cytotoxic assays considering 5 different tumor cell lines and a nontumoral one. These complexes exhibit several important features considered advantageous for developing anticancer agents. Unlike cisplatin analogues, these monofunctional compounds would only coordinate to DNA through a single covalent bond, imposing a different distortive mechanism on the duplex and leading, thus, to a different activity. Moreover, the presence of the ferrocene unit can further modify the mechanism of action and the selectivity of the standard platinum derivatives. To scrutinize the DNA binding mechanism of these novel complexes at molecular level, we have tracked with classical methods the dynamics of a DNA dodecamer duplex interacting with the heteronuclear Fe–Pt complex and compared the results with the distortion induced by cisplatin. These simulations allow us to analyze the distortion of the double strand, placing particular emphasis on the chemical origin of such distortion. These results might help elucidating the differences in the activity of the mono and bifunctional Pt­(II) complex examined and in the design of improved Pt complexes tailored for antineoplastic applications.

## Results and Discussion

The general procedure for the preparation of the monofunctional complexes **1–3** is depicted in Figure 2.A. For the synthesis of these heterobimetallic compounds, the bifunctional complexes *cis*-[PtCl_2_Am_2_] where Am is *N*,*N*-dimethylamine, isopropylamine or ammonia, and *p*-ferrocenylaniline **(L)** were used as starting materials. The reaction takes place in two stages. In the first, the Pt complex is treated with one equivalent of a silver salt in DMF under dark conditions. Then, subsequent treatment with the ligand **L** leads to heterometallic monofunctional complexes **1–3**. Two different silver salts (AgNO_3_ and AgOTf) were employed to modify the counterion of the final products and, thus, their solubility.^41,42^ As a result, triflates **1.b**-**3.b** displayed a better solubility in organic solvents (such as acetone) than nitrate analogues **1.a**-**3.a**. Furthermore, counterions like nitrate could be considered toxic agents, whereas, to the best of our knowledge, there are no toxicological data available for the triflate ion.^43^

**Figure 2:**
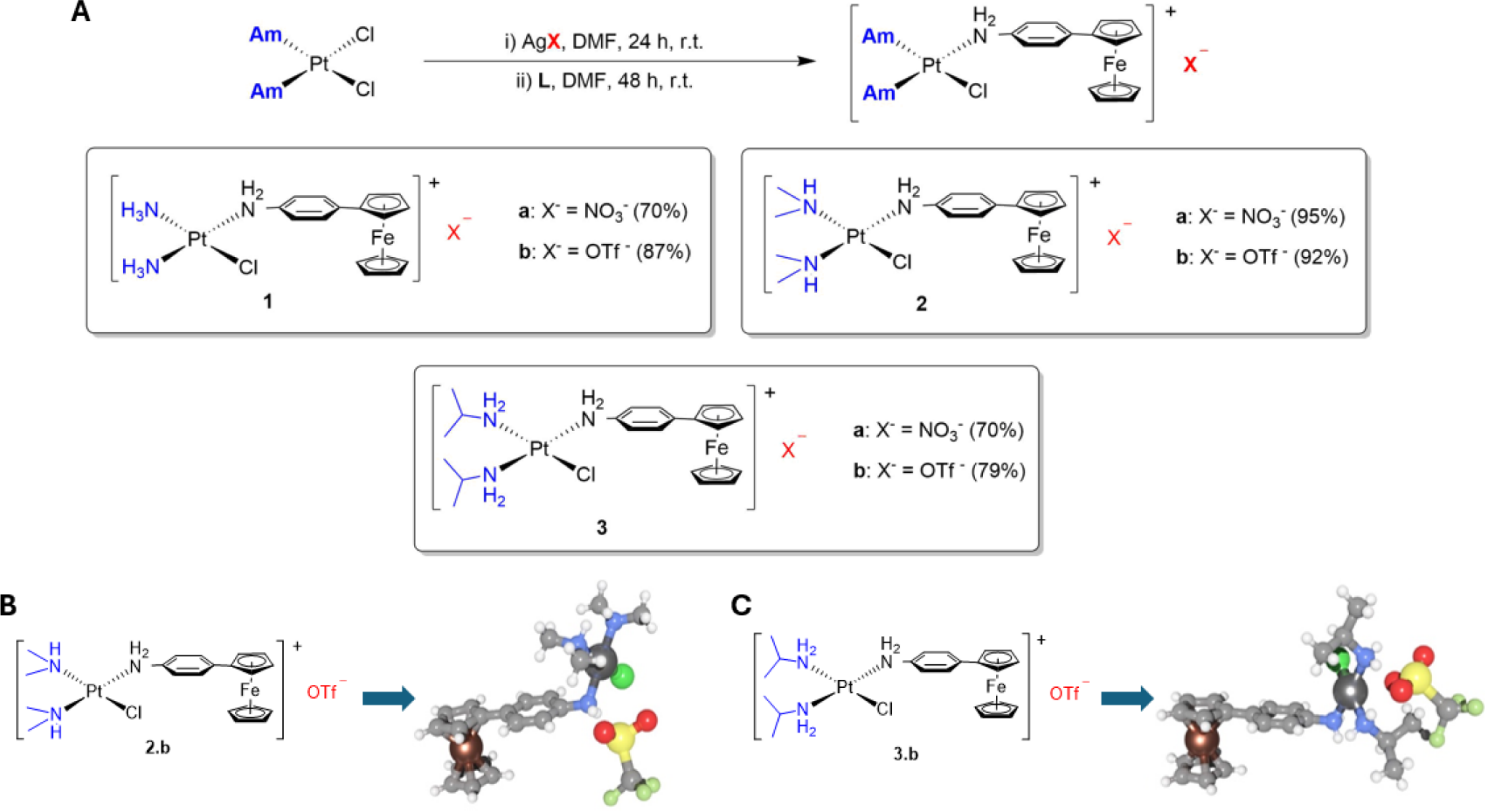
(**A**) Synthesis of heterometallic complexes **1**-**3**. **L** stands for the *p*-ferrocenylaniline ligand and **Am** for the amine. (**B**) Single-crystal X-ray structure of **2.b**. (**C**) Single-crystal X-ray structure of **3.b**.

The formation of the complexes was confirmed by elemental microanalysis, NMR spectrometry, and mass spectrometry. In the ^1^H NMR spectra of **1**-**3**, the protons of the amino group and the aromatic moiety of ligand **L** were strongly deshielded due to the coordination to the Pt­(II) center (Δδ ∼ 2 ppm and ∼0.3–0.7 ppm, respectively; Figures S1,S6,S8,S13,S15 and S20). The same effect was observed in the signals of the ^13^C NMR spectra (Figures S2,S9 and S16). ^195^Pt NMR signals (Figures S3,S10 and S17) were also consistent with a PtClN_3_ environment. The values of molar conductivity in DMF (50–57 S cm^2^ mol^–1^, Table S4) corroborated the 1:1 electrolytic nature of the synthesized monofunctional complexes.^44^

The solid state structure of **2.b** and **3.b** was also determined by single X-ray diffraction (Table S1, Figures 2B,C, S22 and S23). In both complexes, the platinum ion is square planar, with the *cis* bond angles around the platinum center close to the 90° expected value (Tables S2 and S3). In the case of **3.b,** the structure of the triflate anion is highly disordered along the crystal with two possible conformations of its fluorine and oxygen atoms differing in the rotation angle around the C–S bond. This creates big gaps in the molecular packing of this heterometallic complex.

The electrochemical behavior of ligand **L** and the triflates **1.b**-**3.b** was studied in acetone using cyclic voltammetry (CV) and square wave voltammetry (SWV).

The CVs and SWVs (Figures S24–S27) registered with an anodic limit lower than 1 V, showed that, in all cases, the oxidation of the ferrocene moiety is electrochemically and chemically reversible as the *i*_c_/*i*_a_ ratio is practically equal to 1, the Δ*E*_peak_ has a value around 0.074 V (Tables 1 and S5) and the *E*_peak_ are independent of the scan rate. Furthermore, the compounds comply with the Randles–Sevcik equation as there is a linear correlation in the *i*_c_ vs *v* ^1/2^ representation (Figure S28). As reported in Table 1, ferrocene–platinum complexes **1**-**3.b** have similar potential values, greater than their precursor **L** (∼0.13 V) due to the coordination of the *p*-ferrocenylaniline to the Pt­(II), which removes electronic density from the ferrocene moiety.

**Table 1:** Electrochemical Data of **L** and Triflate Complexes **1.b**–**3.b**

	|*i*_c_/*i*_a_|	Δ*E*_p_ (V)	*E*_1/2_ (V)^a^
**L**	0.9	0.074	0.380
**1.b**	1.0	0.073	0.509
**2.b**	1.0	0.075	0.508
**3.b**	1.0	0.074	0.514

a CV half-wave potentials are measured vs SCE in acetone/0.2 M *n*-Bu_4_NPF_6_ solution at a scan rate of 0.100 V s^–1^.

The CVs were repeated, increasing the anodic limit up to 1.5 V (Figure S29). In the case of **L**, along with the peaks that correspond to the Fc/Fc^+^ system (*E*_1/2_ = 0.380 V), a new peak is observed in the direct scan at 1.006 V, with no assigned cathodic peak due to the electrochemical irreversible oxidation of the amino group to form a radical species. This radical undergoes a chemical reaction whose product is reduced at 0.527 V in the reverse scan.^45^ On the other hand, this process does not take place in the heterometallic complexes **1**-**3.b**, because the electron pair of the N donor atom of *p*-ferrocenylaniline is used to coordinate the Pt­(II) ion.

For the cytotoxic studies, DMSO was employed to completely dissolve compounds **1**-**3** and **L**. This solvent is known for reacting with platinum complexes because of the great affinity of the Pt metal toward S.^46^ Despite this, DMSO is the most common solvent for this kind of assays. In order to evaluate the stability of **1.a**-**3.a** in DMSO, we monitored the evolution in time of the ^1^H NMR spectra of these complexes in a DMSO-d_6_/H_2_O solution at room temperature (Figures 3B, S30 and S31). For complexes **2.a** and **3.a,** no changes were observed even after 48 h, whereas **1.a** was found to undergo a slow exchange of the ligand with DMSO (14% after 24 h and 25% after 48 h, Figure 3A).

**Figure 3:**
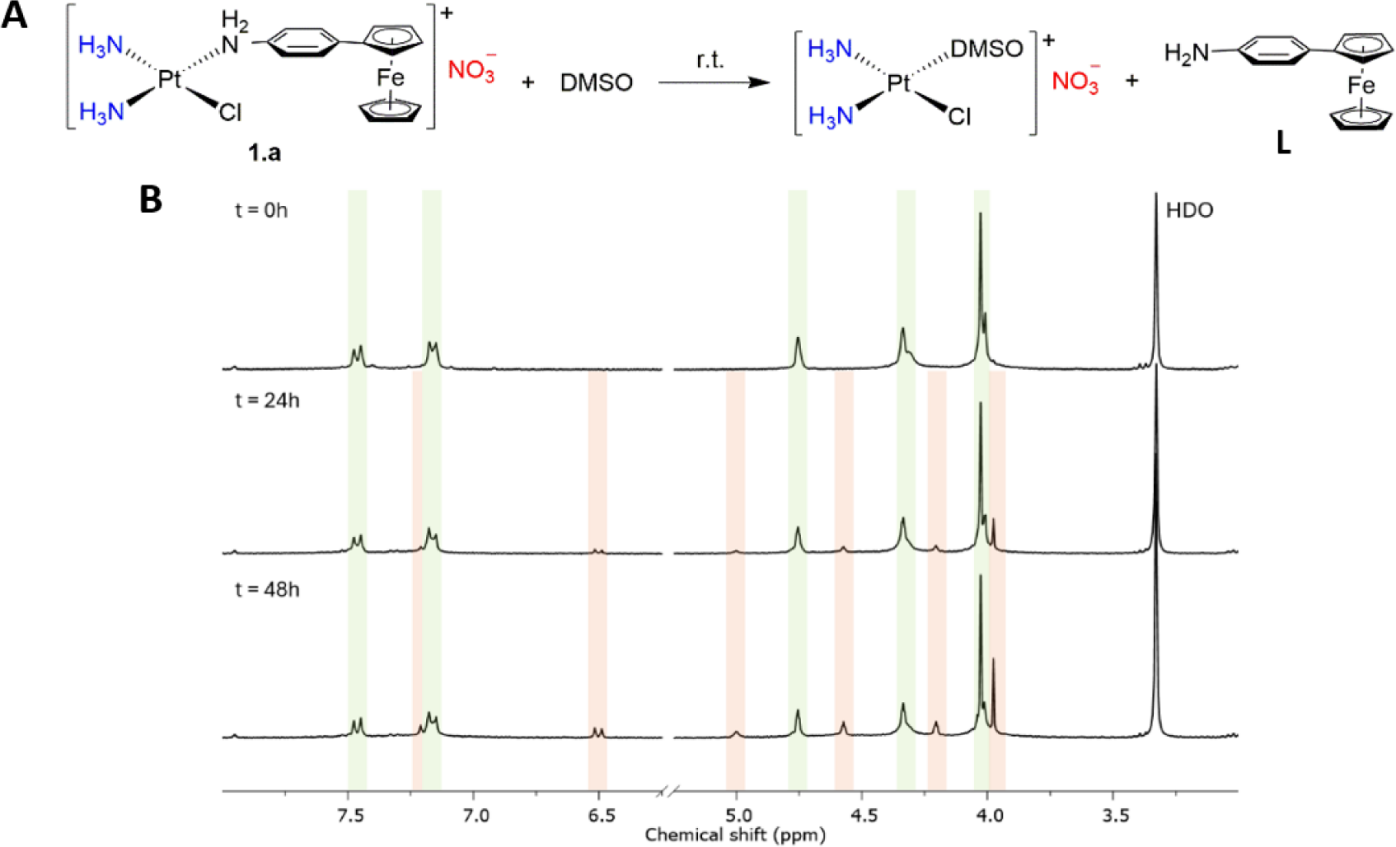
(**A**) Substitution reaction of complex **1.a** in DMSO-*d*_6_/H_2_O. (**B**) ^1^H NMR spectra of the evolution of the reaction of complex **1.a** with DMSO-*d*_6_ over time. The peaks of complex **1.a** and **L** are highlighted in green and orange, respectively.

To evaluate the therapeutic potential of the heterobimetallic complexes synthesized **1–3**, we have conducted cytotoxic activity assessments for these species, alongside for cisplatin (**CPT**) and the ligand *p*-ferrocenylaniline **L**. Notably, the DMSO used as a solvent revealed no toxicity at the maximum concentration used in each assay, indicating that the observed activity can be exclusively attributed to the heterobimetallic platinum–ferrocene complexes.

Five tumor cell lines have been used to study the cytotoxic activity of these compounds, i.e., (i) HeLa cervical cancer, (ii) MDA-MB231 (ER, PR, and HER2 negative) which is commonly used to model late-stage breast cancer and represents a good model of triple-negative breast cancer, (iii) the human breast cancer with estrogen, progesterone, and glucocorticoid receptors MCF-7 which is ″Luminal A″ subtype of noninvasive cell line and highly responsive to chemotherapy, (iv) the colorectal carcinoma HCT-116, and (v) the glioblastoma U87. The cytotoxic activity of these compounds was also evaluated in a nontumoral cell line, the immortalized human epidermal cell line HaCaT, to investigate the possible selectivity of these complexes.

Figure 4 presents the observed cell survival ratio across different cell lines following exposure to heterobimetallic complexes **2.a** and **3.a** and cisplatin (**CPT**), along with *h*_inv_, which allows a straightforward comparison of the theoretical and experimental results. *h*_inv_ can be conceived as a computational parameter derived from molecular dynamics simulations inversely proportional to the oligomer helix distortion upon platination and thus directly comparable to the cell survival to **1–3** complexes. More details on its derivation are provided in Supporting Information. Figure S32 presents the same results for the **2.b** and **3.b** species, whereas Figures S33 and S34 report the same analysis including species **L**, **1.a,** and **1.b**. Among the Pt­(II) complexes tested, cisplatin and the simplest heterobimetallic complexes, **1.a** and **1.b,** generally exhibit the poorest activity together with the ferrocene ligand (**L**) (Figures 4 and S32–S34). In particular, cisplatin only showed activity in HeLa cells and in the nontumoral cell line (HaCaT), whereas the incorporation of the ferrocene ligand to the single-crystalline Pt (II) complexes resulted in decreased toxicity of these complexes in HaCaT cells; see the first panel of Figures 4 and S32–S34. Interestingly, as shown in Figure 4, complexes containing methylated amines, **2.a**, and isopropylated amines, **3.a**, are active in tumoral cell lines, where cisplatin is not, underscoring their selectivity and effectiveness toward tumoral cells. Intriguingly, these monofunctional compounds, and particularly compound **3.b** (Figure S32), demonstrated a dual effect by being active in both MCF-7 and MDA-MB231 cell lines, akin to ferrocifen derivatives currently undergoing clinical trials. Notably, the highest activity of **3.b** was observed in U87 which represents the most aggressive form of brain cancer. Along this study, we have also investigated whether there is any connection between the cytotoxic activity and the counterion present in the complexes. Our results reveal that, depending on the complex, there is either no significant difference in activity between the two salts of the complex or one of the salts is slightly more active. For instance, the nitrate salt of complex **3** (**3.a**, Figure 4) is generally more cytotoxic than its corresponding triflate salt (**3.b**, Figure S32). The opposite is, however, observed for complex **2**, although to a lesser extent. Nevertheless, in nontumoral cells, the nitrate salts (**2.a** and **3.a**, Figure 4) are more active than the triflate salts **(2.b** and **3.b**, Figure S32). In the following, we aim at providing mechanistic insight into the cytotoxic profile of these complexes by examining their interactions with DNA as a target. For this, we will analyze the results obtained from classical molecular dynamics simulations considering the adducts formed between the heterobimetallic complexes **1–3** and a DNA double-strand model. The structure of a crystallized double-stranded 5′-CCTCTGGTCTCC-3′ dodecamer doubly platinated at the two central guanosine monophoshate residues (dG6 and dG7, see Scheme S1) of the same strand of ref. 47 was employed as the initial geometry for the dynamics of unplatinated DNA and of our adducts with complexes **1–3** (platinating the dG6 residue), after removing cisplatin or exchanging cisplatin for our heteronuclear complexes. For the molecular dynamics simulations of the DNA-cisplatin adduct, the crystallized structure was not further modified. The duplex was solvated within a truncated octahedron box of TIP3P^48^ water molecules with a buffer of 12 Å for the DNA strands.

**Figure 4:**
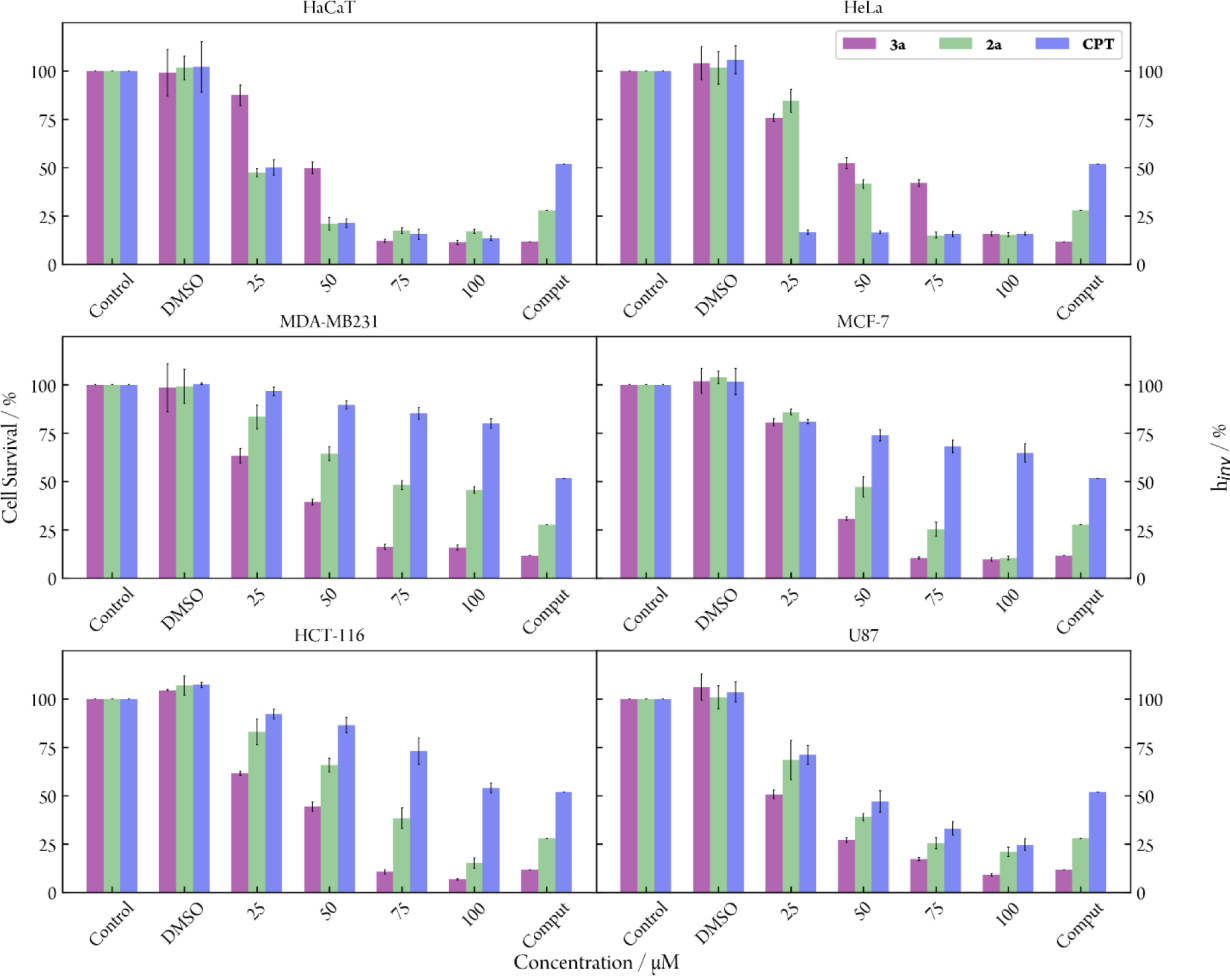
Cell survival of different tumoral and nontumoral (HaCaT) cell lines upon exposure to selected concentrations of the nitrate salts of the complexes **2.a** (green), **3.a** (purple), or cisplatin (**CPT**, blue). The last group of bars in each panel refers to the duplex distortion measured as *h*_inv_, a dimensionless parameter derived from MD simulations defined as the inverse ratio between the average helical bend of the DNA–heterobimetallic complex adduct and that of free DNA (see Supporting Information for details). Although not physically meaningful on its own, *h*_inv_ facilitates a comparison with cell survival trends.

Three independent 500 ns, i.e., 1.5 μs in total, molecular dynamics trajectories were run for the free DNA double strand (ds-DNA) that will be used as a reference, for the DNA cisplatin (ds-DNA+**CPT**) adduct and the adducts of DNA with **1–3** complexes (ds-DNA+**1**, ds-DNA+**2,** and ds-DNA+**3** respectively). All the calculations were performed using PMEMD_CUDA from Amber20.^49^ The convergence of the simulations was ensured through monitoring of the RMSD (Figure S36). Further computational details are provided in the Experimental Section and Supporting Information.

To evaluate the impact of platination with cisplatin and complexes **1–3** in the structure of the DNA double strand model, we considered the bend angle implemented in CURVES+,^50^ which computes the incremental bend in the helical axis at each base pair level. To the same aim, we monitored the distance of key hydrogen bonds along the distortion of the DNA duplex structure. Figures 5 and 6 show the evolution of the cumulative average bend angle (*h*_avg_) and the three hydrogen bond distances of the DNA base pairs undergoing platination or adjacent to the platination spot, dG6-dC19 and dG7-dC18, along the dynamics for each of the models considered.

**Figure 5:**
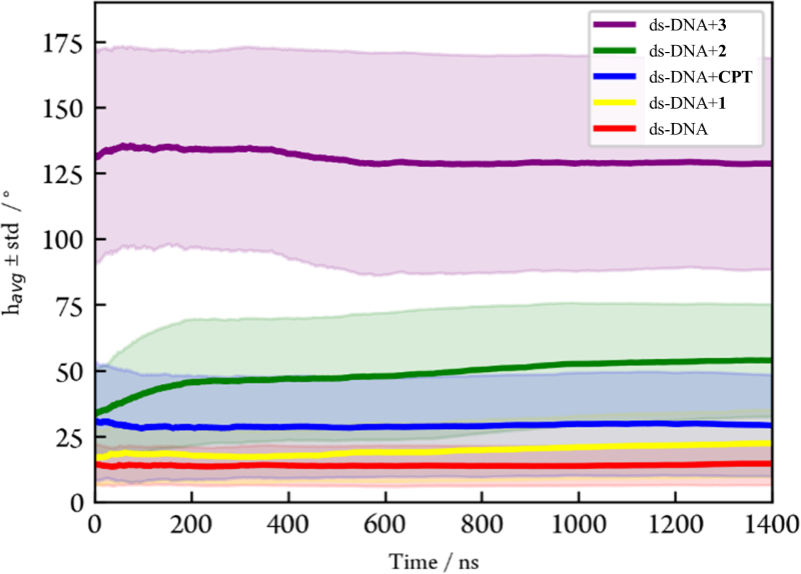
Cumulative average bend angle (solid lines) of the double helix along the simulation for all the models considered, i.e., ds-DNA stands for the free DNA double strand, ds-DNA+**CPT** for the DNA cisplatin adduct, and ds-DNA+**1**, ds-DNA+**2,** and ds-DNA+**3** for the adducts of DNA with **1–3** complexes, respectively. The shadow areas represent the standard deviations calculated for the *h*_avg_ value.

**Figure 6:**
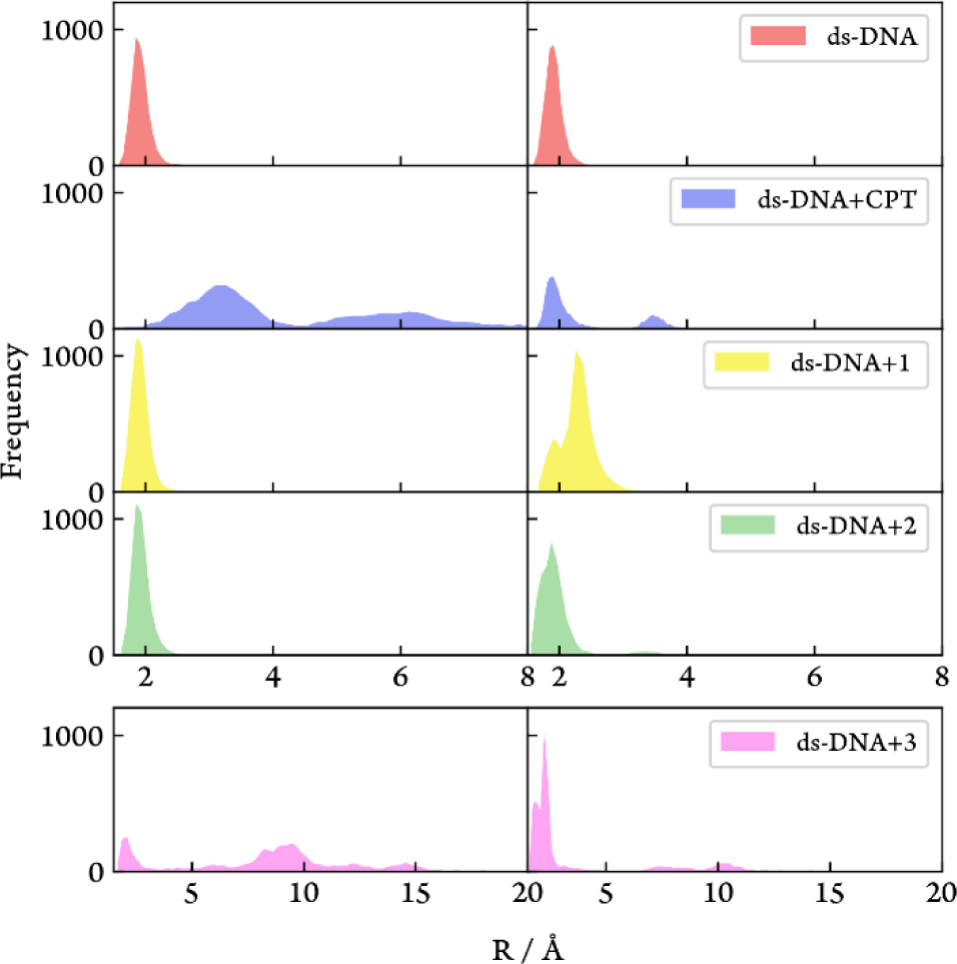
Histogram of the three hydrogen bond lengths for the base pairs dG6-dC19 (left panel) and dG7-dC18 (right panel) for all of the simulations.

As expected, cisplatin (blue curve in Figure 5) presents a higher *h*_avg_ value of 29 ± 19° than unplatinated DNA (*h*_avg_ = 15 ± 8°, red curve in Figure 5). The larger mean value obtained for cisplatin reveals a clear distortion of the helix provoked by the platinum complex, which is at the heart of the well-known cytotoxicity of cisplatin. Other anomalous geometrical parameters confirm the significant distortion of the model double strand upon the coordination of **CPT**. In fact, the cumulative tilt angle for the platinated base pairs or adjacent to the coordination sites for the ds-DNA+**CPT** adduct is significantly altered compared with that of the free duplex (Figure S37). The coordination of **CPT** also importantly affects the cumulative opening angle between the complementary nucleobases directly bonded to **CPT** and the ones occupying the adjacent positions (Figure S38). A careful examination of the structure of the dodecamer duplex after platination with **CPT** reveals that 4 out of the 6 hydrogen bonds originally pairing the two platinated guanines with the complementary cytosines of the opposite strand (dG6-dC19 and dG7-dC18) become either stretched or broken along the trajectory (Figure 6).

The disruption of the hydrogen bonds in the base pairs undergoing platination is consistent with the NMR data from the literature.^51^ In fact, cisplatin induces a torsion of the duplex strong enough (the calculated force constant for the dG-Pt-dG bond angle is 177 kcal·mol^–1^·rad^–2^) to break or at least significantly stretch some of the hydrogen bonds in which the platinated guanines are involved, consistent with the idea that cytotoxicity of cisplatin relies on the distortion of the helix originated from the double platination and the covalence of these bonds. Some representative geometries of the DNA+**CPT** complexes for the three clusters found along one of the trajectories ran are shown in Figure 7.

**Figure 7:**
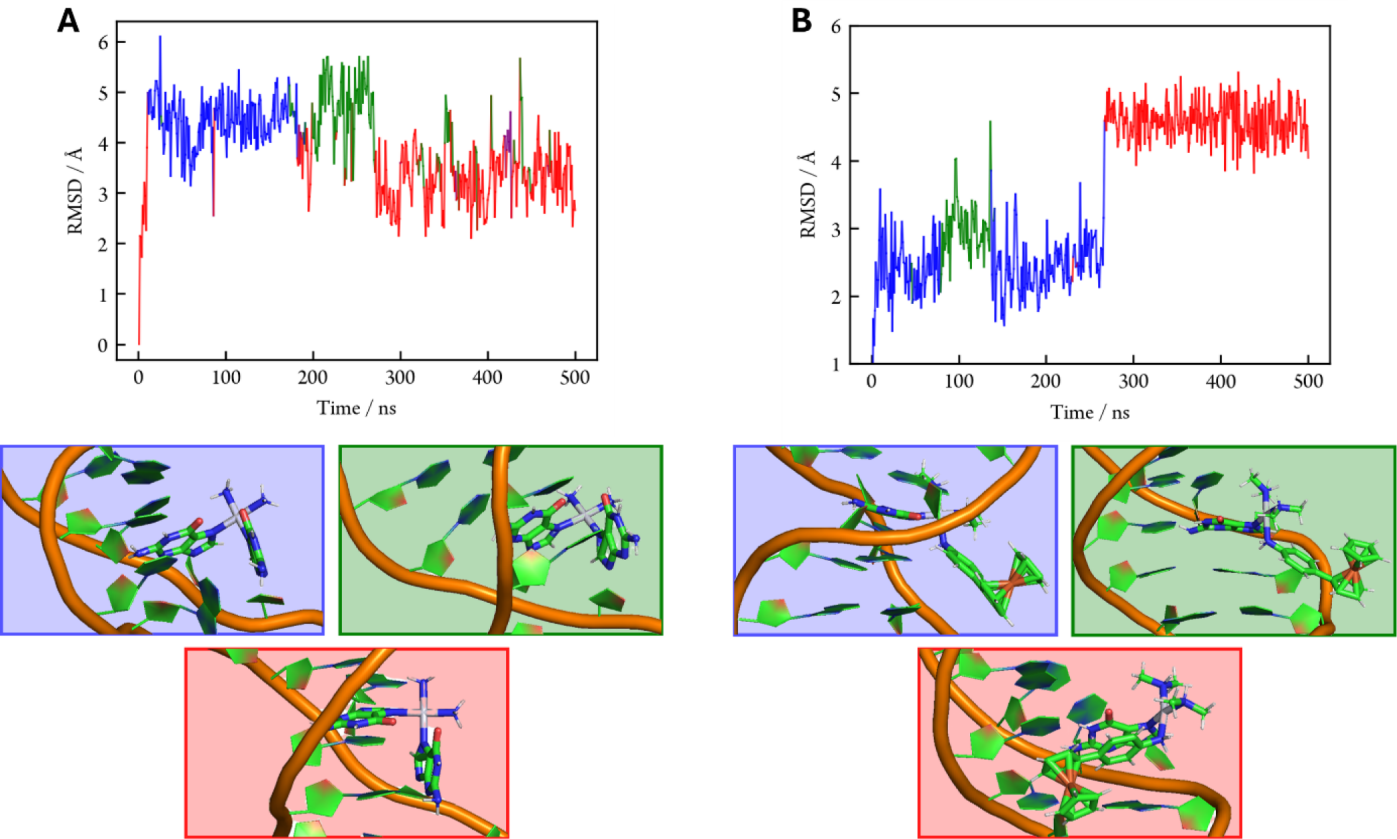
Evolution of the RMSD along a representative trajectory of (**A**) ds-DNA+**CPT** and (**B**) ds-DNA**+2** displaying the relevant clusters: cluster 1 (blue), cluster 2 (green), and cluster 3 (red) and selected geometries.

Consistently with the weak activity registered for this species in the cytotoxicity assays, a smaller distortion of the double helix is observed for the simplest heterobimetallic complex **1**, presenting nonalkylated amines, upon coordination to the dG6 of the model duplex (*h*_avg_ = 22 ± 13°, yellow curve in Figure 5). The monitoring of the hydrogen bond distances for the dG6-dC19 and dG7-dC18 base pairs reveals no substantial changes in the hydrogen bond distances upon ds-DNA+**1** adduct formation, although a small elongation of two hydrogen bonds from the dG7-dC18 base pair is registered (Figure 6). The reduced number of affected hydrogen bonds and the smaller degree of hydrogen bond distortion induced by **1** compared to **CPT** is consistent with the smaller deformation produced by the former (Figure 5).

The heteronuclear complex **2**, which presents methylated amino groups, induces a greater distortion on the DNA duplex (*h*_avg_ value of 54 ± 21°, green curve in Figure 5) compared to **CPT** and complex **1**. Coordination of complex **2** to the duplex also produces important changes in the cumulative roll and propeller angles, especially for the late stages of the propagations (Figure S39), affecting consecutive pairs of nucleobases adjacent to the coordination spot and further away. Upon inspection of the hydrogen bond pattern of the nonplatinated guanosine residue (dG7) within the DNA+**2** adduct (Figure 6), we find that only one hydrogen bond becomes either elongated at some stage of the simulation.

The detailed tracking of the dynamics for the DNA**+2** adduct revealed an unexpected event occurring 250 ns after the simulations were initiated. Interestingly, the three trajectories disclose a conspicuous decrease in the distance between ferrocene and the platinated dG6 residue. This change in the Fe-dG6 distance arises from the rotation of the ferrocene moiety around the phenyl-cyclopentadienyl bond axis. The results for one of the trajectories are reported in Figure 8. Figure 7 collects some representative geometries of the ds-DNA**+2** complex for the 3 clusters identified along the same trajectory analyzed in Figure 8. The same analysis for the other two trajectories can be found in Figure S40, which undergo rotation of the ferrocene moiety at earlier stages of the propagation.

**Figure 8:**
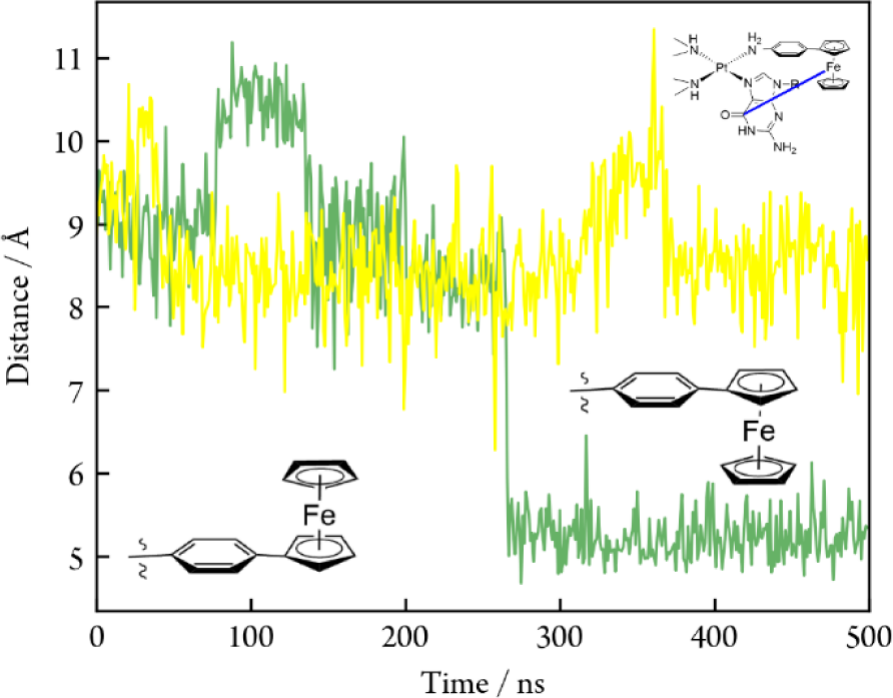
Evolution of the distance (in Angstroms) between the Fe atom from the ferrocene unit and the C6 atom from the dG6 residue along a selected trajectory of ds-DNA+**1** (yellow) and ds-DNA+**2** (green).

To understand the implications of the rotation of the ferrocene, we monitored the evolution of the noncovalent interactions, NCI, along this trajectory (further computational details are provided in the Supporting Information Section S1). Interestingly, our NCI analysis, see Figure S41a, reveals an increase by 13% of the integrated volumes of the reduced density gradient (s) after the rotation of the ferrocene (vertical blue line) that is ascribed to the creation of new van der Waals interactions (weak attractive interactions) promoted by the change in the conformation of the metallocene. In fact, this increase in the weak attractive interactions coincides with the intercalation of ferrocene within the duplex and its stacking with the dC19 residue (Figure S42). Moreover, a more comprehensive analysis of the NCI based on the reduced density gradients plots and employing this time SCF electron densities revealed that besides the creation of new van der Waals interactions, two new hydrogen bonds involving the platinated guanine dG6 were established after the rotation of the ferrocene moiety (Figure S43), the first with its complementary nucleobase dC19, which was broken along the first stages of the coordination to the Pt­(II) complex and the second with the cytosine from the contiguous base pair (dC18) (Figure S44).

An analogous analysis along the dynamics for the adducts with the nonalkylated complex **1** reveals that the ferrocene moiety also undergoes rotations around the phenyl-cyclopentadienyl bond for this complex but that it immediately flips back to its original position (Figures 8 and S45). Consistently with this, no increase in the total number of van der Waals interactions is registered for this complex (Figure S41b). Considering that the only difference between the two Pt­(II) complexes examined lies in the substitution of the amino ligands, we infer that the steric constrain induced by the methyl groups in complex **2** enables the access to a new conformational space in which the ferrocene moiety can intercalate the DNA. The creation of two new hydrogen bonds between the platinated dG6 and the residues dC18 and dC19 together with the establishment of new stacking interactions between the ferrocene moiety and dC19 would stabilize the new conformation for the adduct with complex **2**. Our calculations put the focus on the methyl substituents of the amino group of the heterometallic complex as key agents for the cytotoxicity in these species.

Finally, the value of the bend angle for the duplex adduct with complex **3**, ds-DNA+**3**, registered at the end of the propagations amounts to 129 ± 40° (purple curve in Figure 5), notably surpassing the values calculated for any of the other Pt­(II) complexes considered. For complex **3**, the cumulative tilt angle defined by the nucleobase coordinated to the complex and the adjacent base pair undergoes important changes along the propagation (Figure S46). The scrutiny of the structural evolution of the adduct along the trajectories run for ds-DNA+**3** reveals a different binding mode mechanism for this complex, in which the ferrocene ligand does not actively participate. In fact, the steric repulsion brought by the isopropyl moieties of complex **3** upon coordination to dG6 displaces a nucleotide out from the pairing cavity toward the solvent (Figure S47). However, no particular trend is found among the trajectories examined. As a matter of fact, the nucleotide that is expelled varies depending on the trajectory, i.e., dC18, dC19, and dA20 were found to be displaced from their original location, affecting, thus, to different extents the hydrogen bond distances between the two central base pairs of the duplex (Figures 6 and S48). The platinated dG6-dC19 base pair in the trajectories of the ds-DNA+**3** adduct presents hydrogen bond distances that may reach 10 or even 15 Å, which are related to the expulsion of the dC19 residue from the pairing region. Hence, while a direct relationship can be observed between the number of broken/stretched hydrogen bonds from the Watson–Crick pattern of the platinated guanines for the **1, 2,** and **CPT** complexes and the distortion induced in the DNA duplex, complex **3** does not conform to that trend. In fact, the significant steric repulsion created by the voluminous isopropyl moieties pushes other nucleotides out of the inner pairing cavity of DNA, resulting in a different DNA distortion mechanism, sometimes affecting regions of the duplex that are distant from the platination spot. Interestingly, there is a nice and clear correlation between the degree of duplex distortion predicted by the classical molecular dynamics simulations (see bars associated to the “Comput” label in Figure 4) and the cell survival recorded in the cytotoxic assays (rest of the bars in Figure 4).

## Conclusion

This study presents the synthesis and comprehensive characterization of a novel family of heterobimetallic Pt­(II)-ferrocene complexes. These new compounds, derived from bifunctional complexes *cis*-[PtCl_2_Am_2_] (where Am = NH­(CH_3_)_2_ or NH_2_CH­(CH_3_)_2_) and *p*-ferrocenylaniline (**L**), exhibit remarkable cytotoxic activity against the studied tumoral cell lines such as HeLa, HCT-116, MCF-7, MDA-MB231, and U87, surpassing that of cisplatin for all cases except HeLa, whereas the amino complexes were generally less active. Also, complexes **2.b** and specially **3.a** and **3.b** at low concentrations (25 and 50 μM) demonstrate a notable lack of cytotoxicity against the nontumoral HaCaT cell line, showcasing promising selectivity properties. Subtle differences in the cytotoxicity were observed between complexes carrying the counterions NO_3_^–^ (**a**) and TfO^–^ (**b**), with the nitrate showing a slightly higher cytotoxic behavior for **3**. To gain insight into their mechanism of action at the molecular level, comprehensive classical molecular dynamics simulations were conducted considering DNA as the target. These simulations revealed a correlation between the cytotoxicity and the simulated cumulative bend angle. An analysis of the molecular dynamics of the DNA-complex adducts unveils different distortion mechanisms of the DNA duplex. Upon double coordination to DNA, **CPT** induces the breaking or at least elongation of 4 hydrogen bonds established by the platinated guanines, leading to significantly rearranged structures of the duplex in the adduct. In the case of monofunctional complexes, the bulkiness of the substituents of the amino groups was found to be closely related to the ability of the Pt­(II) complex and/or the DNA components to explore new conformational spaces, resulting in highly distorted structures of the duplex. The distortion mechanism involves either the rotation of the ferrocene moiety (compound **2**), facilitating new hydrogen bonds between originally non-interacting base pairs and new weak attractive stacking interactions between the Pt­(II) complex and neighboring nucleobases, or the rotation of diverse nucleotides toward the solvent environment (compound **3**).

The promising findings of this study pave the way for the characterization of a new class of hybrid drugs combining Pt­(II) amines and ferrocene, offering an alternative to bifunctional compounds with enhanced *in vitro* efficacy. Additionally, the mechanistic insights provided by classical molecular dynamics simulations offer valuable guidance for the design of future compounds with improved therapeutic properties.

## Experimental Section

### Chemical Reagents and Instruments

All of the reagents and solvents were analytical grade and used as received without further purification. Silica gel Si 60 (40–63 μm; Merck) was used for column chromatographic purifications. Infrared spectra were recorded on a PerkinElmer 100 FT-IR spectrometer using KBr pellets, an ATR (attenuated total reflectance), or a Nujol suspension with CsI windows. Elemental analyses were performed in a LECO CHNS-932 elemental analyzer. ^1^H, ^13^C, and ^195^Pt NMR spectra, as well as two-dimensional spectra, were recorded on a Bruker Avance 300 and a Bruker Avance III-HD Nanobay 300 spectrometer from the NMR Laboratory, SIdI, Universidad Autónoma de Madrid, Spain. Chemical shifts were reported in parts per million (δ) with reference to residual solvent resonances for ^1^H and ^13^C NMR (CDCl_3_: δ ^1^H = 7.26 ppm, δ ^13^C = 77.2 ppm; acetone-d_6_: δ ^1^H = 2.05 ppm, δ ^13^C = 206.3/29.8 ppm; DMSO-d_6_: δ ^1^H = 2.50 ppm, δ ^13^C = 39.5 ppm). Multiplicities are indicated as s, singlet; d, doublet; t, triplet; pt, pseudotriplet; sept., septuplet; and br s, broad singlet. ^195^Pt NMR spectra were referenced externally using 1.0 M Na_2_PtCl_6_ in D_2_O. The electrospray ionization (ESI) mass spectra were recorded on a MAXIS II spectrometer, while FAB mass spectra were obtained by using a VG AutoSpec mass spectrometer with *m*-nitrobenzyl alcohol (*m*-NBA) as the matrix. Samples were prepared in dichloromethane solutions. Conductivity measurements were carried out in 10^–3^ M DMF solutions of the monofunctional complexes using a Crison EC-meter GLP 31 conductivity cell.

### Synthesis of the Precursors

The bifunctional complexes *cis*-[PtCl_2_{NH­(CH_3_)_2_}_2_], *cis*-[PtCl_2_{NH_2_CH­(CH_3_)_2_}_2_], and cisplatin were synthesized following the procedure described in the literature.^52,53^ The ligand NH_2_(*p*-C_6_H_4_)­Fc (**L**) was prepared according to the previously reported method.^54^

### General Procedure for the Preparation of the Heterometallic Complexes

In a 10 mL round-bottomed flask, a solution of 1.0 equiv of the bifunctional platinum­(II) complex in 3 mL of DMF was prepared, and 1.0 equiv of AgNO_3_ or AgOTf was added. The mixture was stirred at room temperature without light for 24 h. The precipitated AgCl was separated by filtration through celite, and 1.0 equiv of the ligand NH_2_(*p*-C_6_H_4_)­Fc (**L**), was added to the filtrate. The mixture was stirred at room temperature without light for 48 h. The solvent was evaporated under reduced pressure and 1.2 mL of acetone (**1.a**, **1.b**, **2.a,** and **3.a**) or 14 mL of ether (**2.b** and **3.b**) was added. The solution was kept in the dark at 0–4 °C. The solid was separated by filtration and dried under vacuum.

*cis*-[PtCl­(NH_3_)_2_{NH_2_(*p*-C_6_H_4_)­Fc}]­NO_3_ (**1.a**). Yield: 70% (0.1041 g, 0.173 mmol). Anal. Calcd for C_16_H_21_FeN_4_ClPtO_3_: C, 31.83; H, 3.51; N, 9.28. Found: C, 32.40; H, 3.54; N, 9.20. ^1^H NMR (DMSO-d_6_, 300 MHz): δ 4.03 (s, 5H, H_8_), 4.01, 4.31 (br s, 6H, H_b_ or H_c_), 4.33 (pt, ^3^ *J*_H,H_ = 1.9 Hz, 2H, H_7_), 4.72 (pt, ^3^ *J*_H,H_= 1.9 Hz, 2H, H_6_), 7.16 (br s, 2H, H_a_), 7.16 (d, ^3^ *J*_H,H_ = 8.4 Hz, 2H, H_2_), 7.49 (d, ^3^ *J*_H,H_ = 8.4 Hz, 2H, H_3_). ^13^C NMR (DMSO-d_6_, 75 MHz): δ 66.2 (C_6_), 68.8 (C_7_), 69.3 (C_8_), 84.6 (C_5_), 121.7 (C_2_), 125.9 (C_3_), 135.6 (C_4_), 139.9 (C_1_). ^195^Pt NMR (DMSO-d_6_, 64 MHz): δ −2340. IR (ATR, cm^–1^): ν­(N–H) 3289, 3215; δ­(N–H) 1526; ν­(C_ar_–C_ar_) 1599, 1350, 1106; ν­(NO_3_) 1327; π­(C_Fc_–H) 888, 837; δ­(C_ar_–H) 1000, 813; π­(NO_3_) 826. IR (Nujol, CsI, cm^–1^): ν­(Fe–Cp) 513, 503; δ_asym_(Cp–Fe–Cp) and ν­(Pt–N) 488, 437; ν­(Pt–Cl) 332. MS (ESI^+^): *m*/*z* 277.1 [M–PtCl­(NH_3_)_2_]^+^, 342.0 [M+DMSO–Fc­(*p*-C_6_H_4_)­NH_2_]^+^, 542.0 [M^+^].

*cis*-[PtCl­(NH_3_)_2_{NH_2_(*p*-C_6_H_4_)­Fc}]­OTf (**1.b**). The precipitated solid, obtained when acetone was added, was filtered, and the solvent of the filtrate was evaporated under reduced pressure. The resulting solid was the desired monofunctional complex. Yield: 87% (0.1968 g, 0.286 mmol). The spectroscopic data are the same as in **4.a** with some differences in the IR bands. IR (ATR, cm^–1^): ν_as_(SO_3_) 1271, ν_s_(CF_3_) 1239, ν_as_(CF_3_) 1160, ν_s_(SO_3_) 1030, δ_s_(CF_3_) 760, δ_s_(SO_3_) 637, δ_as_(CF_3_) 579. Anal. Calcd for C_17_H_21_FeN_3_ClPtSF_3_O_3_: C, 27.34; H, 2.83; N, 5.63. Found: C, 27.82; H, 2.98; N, 5.67.

*cis*-[PtCl­{NH­(CH_3_)_2_}_2_{NH_2_(*p*-C_6_H_4_)­Fc}]­NO_3_ (**2.a**). Yield: 95% (0.1606 g, 0.245 mmol). Anal. Calcd for C_20_H_29_FeN_4_ClPtO_3_: C, 36.40; H, 4.43; N, 8.49. Found: C, 36.99; H, 4.58; N, 8.30. ^1^H NMR (DMSO-d_6_, 300 MHz): δ 2.50 (m, H_9_ and H_10_), 4.01 (s, 5H, H_8_), 4.35 (s, 2H, H_7_), 4.77 (s, 2H, H_6_), 5.69 (br s, 1H, H_b_ or H_c_), 5.79 (br s, 1H, H_b_ or H_c_), 6.50 (br s, 1H, H_a_), 7.25 (br s, 1H, H_a_), 7.34 (d, ^3^ *J*_H,H_ = 8.0 Hz, 2H, H_2_), 7.50 (d, ^3^ *J*_H,H_ = 8.0 Hz, 2H, H_3_). ^13^C NMR (DMSO-d_6_, 75 MHz): δ 43.2, 43.3 (C_9_ and C_10_), 66.2 (C_6_), 68.9 (C_7_), 69.4 (C_8_), 84.1 (C_5_), 123.0 (C_2_), 126.3 (C_3_), 136.4 (C_4_), 139.5 (C_1_). ^195^Pt NMR (DMSO-d_6_, 64 MHz): δ −2407. IR (ATR, cm^–1^): ν­(N–H) 3200, 3122; δ­(N–H) 1530; ν­(C_ar_–C_ar_) 1460, 1405, 1345, 1103; ν­(NO_3_) 1388; π­(C_Fc_–H) 889; δ­(C_ar_–H) 1007, 815; π­(NO_3_) 832; δ­(NO_3_) 711. IR (Nujol, CsI, cm^–1^): ν­(Fe–Cp) 508; δ_asym_(Cp–Fe–Cp) and ν­(Pt–N) 488, 446; ν­(Pt–Cl) 333. MS (ESI^+^): *m*/*z* 597.1 [M^+^].

*cis*-[PtCl­{NH_2_CH­(CH_3_)_2_}_2_{NH_2_(*p*-C_6_H_4_)­Fc}]­OTf (**2.b**). Yield: 92% (0.1932 g, 0.259 mmol). The spectroscopic data are the same as **2.a** with some differences in the IR bands. IR (ATR, cm^–1^): ν_as_(SO_3_) 1282, ν_s_(CF_3_) 1246, ν_as_(CF_3_) 1162, ν_s_(SO_3_) 1030, δ_s_(CF_3_) 762, δ_s_(SO_3_) 638, δ_as_(CF_3_) 576. Anal. Calcd for C_21_H_29_FeN_3_ClPtSF_3_O_3_: C, 33.77; H, 3.91; N, 5.63. Found: C, 34.47; H, 4.04; N, 5.58.

*cis*-[PtCl­{NH_2_CH­(CH_3_)_2_}_2_{NH_2_(*p*-C_6_H_4_)­Fc}]­NO_3_ (**3.a**). Yield: 70% (0.1257 g, 0.184 mmol). Anal. Calcd for C_22_H_33_FeN_4_ClPtO_3_: C, 38.41; H, 4.83; N, 8.14. Found: C, 38.29; H, 4.84; N, 8.25. ^1^H NMR (DMSO-d_6_, 300 MHz):δ 1.15, 1.22 (d, ^3^ *J*_H,H_ = 6.4 Hz, 12H, H_11_ and H_12_), 3.15 (m, 2H, H_9_ and H_10_), 3.99 (s, 5H, H_8_), 4.35 (pt, ^3^ *J*_H,H_ = 1.9 Hz, 2H, H_7_), 4.79 (pt, ^3^ *J*_H,H_ = 1.9 Hz, 2H, H_6_), 4.83 (br s, 1H, H_b_ or H_c_), 5.02 (br s, 1H, H_b_ or H_c_), 7.10 (br s, 2H, H_a_), 7.33 (d, ^3^ *J*_H,H_ = 8.3 Hz, 2H, H_2_), 7.49 (d, ^3^ *J*_H,H_ = 8.3 Hz, 2H, H_3_). ^13^C NMR (DMSO-d_6_, 75 MHz): δ 23.1 (C_11_ and C_12_), 47.1, 48.2 (C_9_ and C_10_), 66.2 (C_6_), 68.9 (C_7_), 69.4 (C_8_), 84.1 (C_5_), 123.2 (C_2_), 125.9 (C_3_), 136.2 (C_4_), 139.6 (C_1_). ^195^Pt NMR (DMSO-d_6_, 64 MHz): δ −2440. IR (ATR, cm^–1^): ν­(N–H) 3236, 3134; ν­(C_sp3_–H) 2973; δ­(N–H) 1527; ν­(C_ar_–C_ar_) 1460, 1394, 1352, 1105; ν­(NO_3_) 1374; π­(C_Fc_–H) 889; δ­(C_ar_–H) 999, 809; π­(NO_3_) 840; δ­(NO_3_) 727. IR (Nujol, CsI, cm^–1^): ν­(Fe–Cp) 508; δ_asym_(Cp–Fe–Cp) and ν­(Pt–N) 490, 435; ν­(Pt–Cl) 340. MS (FAB): *m*/*z* 277.1 [M–PtCl­{NH_2_CH­(CH_3_)_2_}_2_]^+^, 625.3 [M^+^].

*cis*-[PtCl­{NH_2_CH­(CH_3_)_2_}_2_{NH_2_(*p*-C_6_H_4_)­Fc}]­OTf (**3.b**). Yield: 79% (0.1041 g, 0.173 mmol). The spectroscopic data are the same as **3.a** with some differences in the IR bands: IR (ATR, cm^–1^): ν_as_(SO_3_) 1281, ν_s_(CF_3_) 1233, ν_as_(CF_3_) 1169, ν_s_(SO_3_) 1028, δ_s_(CF_3_) 762, δ_s_(SO_3_) 640, δ_as_(CF_3_) 571. Anal. Calcd for C_22_H_33_FeN_4_ClPtO_3_: C, 35.65; H, 4.29; N, 5.42. Found: C, 35.57; H, 4.55; N, 5.42.

### Crystallographic Data

The heterometallic complexes **2.b** and **3.b** were structurally characterized by single-crystal X-ray diffraction. Suitable orange and orange-yellow crystals of **2.b** and **3.b,** respectively, were coated with mineral oil and mounted on MiTeGen MicroMounts. The samples were transferred to a Bruker D8 KAPPA Series II diffractometer with an APEX II area-detector system equipped with graphite-monochromated Mo Kα radiation (λ = 0.71073 Å). After data collection and integration with the Bruker SAINT software package,^55^ absorption corrections were applied using the Multiscan method (SADABS).^56^ The software package Bruker SHELXTL^57^ was used for space group determination, structure solution, and refinement. The space group determination was based on a check of the Laue symmetry, and systematic absences were confirmed using a structure solution. The structures were solved by direct methods (SHELXT version 2018/2), completed with different Fourier syntheses, and refined with full-matrix least-squares using SHELXL-2019, minimizing Σ *w*(*F*_o_^2^ – *F*_c_^2^)^2^.^58,59^ All non-hydrogen atoms were refined with anisotropic displacement parameters. The hydrogen atom positions were calculated geometrically and were allowed to ride on their parent carbon or nitrogen atoms with fixed isotropic *U* values. All scattering factors and anomalous dispersion factors are listed in the SHELXTL 6.10 program library. The crystal structures of the complexes **2.b** and **3.b** have been deposited at the Cambridge Crystallographic Data Center with deposition numbers 2361678 and 2361679.

### Electrochemical measurements

Cyclic voltammetric (CV) and square wave voltammetric (SWV) experiments were recorded on an Autolab PGSTAT302F potentiostat. The supporting electrolyte used was tetra-*n*-butylammonium hexafluorophosphate, *n*-Bu_4_NPF_6_, which was purified by recrystallization from ethanol and dried under vacuum at 60 °C. The supporting electrolyte concentration was 0.2 M. A conventional three-electrode cell connected to an atmosphere of prepurified nitrogen was used. The counter electrode was a coiled Pt wire, and the reference electrode was a BASi saturated calomel electrode (SCE). All experiments were performed using a platinum-disk working electrode (*A* = 0.020 cm^2^) (Bioanalytical Systems). The working electrodes were polished on a Buehler polishing cloth with Metadi II, rinsed thoroughly with purified water and acetone, and allowed to dry. All potentials were referenced to the SCE electrode. Under our conditions, the ferrocene redox couple [FeCp_2_]^0/+^ is 0.476 V vs SCE in acetone 0.2 M *n*-Bu_4_NPF_6_. Solutions were 10^–3^ M in the redox-active species. The solutions for the electrochemical experiments were purged with nitrogen and kept under an inert atmosphere throughout the measurements. Square wave voltammetry (SWV) was performed using a frequency of 10 Hz.

### Cell Culture Studies

#### Cell Culture

HeLa, MCF-7, MDA-MB-231, HCT-116, and U87 MG cell lines were obtained from the American Type Culture Collection (ATCC, USA) and have been used as tumor cell models. HaCaT cell line was obtained from Cytion (CLS Cell Lines Service GmbH, Germany) and has been used as a nontumoral cell model. HeLa, HaCaT, MCF-7, MDA-MB-231, and U87 MG cell lines were grown in Dulbecco’s Modified Eagle’s Medium (DMEM, Cytiva, UT, USA) supplemented with fetal calf serum (FCS, 10%, Gibco) and 0.5% of antibiotics (penicillin G [10,000 U/mL] and streptomycin sulfate [10,000 mg/mL] (Thermo Fisher Scientific (MA, USA)). HCT-116 cells were cultured in McCoy’s 5A medium (Cytiva, UT, USA) adding the same supplements (serum and antibiotics) as above. DMEM and McCoy’s 5A medium supplemented with FCS and antibiotics will be referred to as the complete medium. Cells were grown in a Thermo FORMA Direct Heat cell incubator (Thermo Scientific), with a 5% CO_2_ atmosphere, a 95% relative humidity, and a constant temperature of 37 °C. For the cytotoxicity experiments, cells were plated on 24-well plates.

#### Administration of Compounds

Stock solutions of the corresponding compounds (**L**, **1**-**3,** and cisplatin) were prepared in DMSO (Panreac). The work solutions were obtained by dissolving the compounds in a complete medium. The final concentration of DMSO was always lower than 0.5% (v/v), and the lack of toxicity of this solvent for the cells was also tested and confirmed. All treatments were performed by incubating the different compounds for 24 h with the different cell lines when the cultures reached about 60–70% confluence.

#### MTT Viability Assay

Cell viability was documented by the MTT assay.^60^ 24 h after appropriate treatments, 3-(4,5-dimethylthiazol-2-yl)-2,5-diphenyltetrazolium bromide (MTT) solution was added to each well at a concentration of 0.1 mg/mL, and plates were incubated at 37 °C for 2–3 h. The resulting formazan crystals were dissolved by the addition of DMSO, and the absorbance was measured at 542 nm. The results were expressed as the cell survival percentage of the control (cell survival (%) = (mean OD treated cells/mean OD value of control cells) × 100%).

### Computational Details

The MD production simulations were preceded by a standard protocol consisting of energy minimization, heating, and equilibration. First the positions of the hydrogen atoms, then the solvent molecules, and finally the whole system were optimized back-to-back. For the three minimizations, 1000 steps were considered using a steepest descent algorithm, which was then switched to a conjugate gradient algorithm. Then, the system was heated from 100 to 300 K using the Langevin thermostat with an integration step of 0.5 fs. After heating, a set of five equilibration runs were performed in which progressive weaker restraints were applied to the Pt-DNA complex. In the last steps, the ensemble was switched from an NVT to an NPT. The bonds involving hydrogen atoms were restrained using SHAKE.^61^ Three independent 500 ns molecular dynamics trajectories were run for each system. All the calculations were performed using PMEMD_CUDA from Amber20.^49^

Some properties of the system, such as the distortion of the double helix, were measured by intercalating equivalent frames of three independent trajectories to ensure the convergence of the properties. However, the monitoring of other properties as the evolution of the hydrogen bond distances or the noncovalent interactions was specifically analyzed for each trajectory.

The convergence analysis, clustering analysis, and the atomic displacements of the dynamics were studied using Cpptraj.^49^ The first 100 ns of each trajectory were discarded when analyzing the distortion of the DNA duplex to exclude the stabilization steps. The distortion of the helix was considered using the cumulative average value of the helical bend parameter, *h*_avg_, and its standard deviation at each iteration of the trajectory from the program Curves+,^62^ as well as other geometric descriptors as the opening and tilt of pairs of residues.

For the analysis of the noncovalent interactions, the NCIPlot4.0 ^63,64^ code was used. In this analysis, the noncovalent interactions within the whole system, i.e., the dodecamer and the different heterobimetallic drugs, were calculated along the whole trajectory. The strength and the nature of the noncovalent interactions were characterized based on the magnitude of the reduced density gradient and the sign of the second eigenvalue of the Hessian. This analysis was performed using promolecular densities. An additional NCI analysis was performed using SCF (ωB97XD/def2-TZVPP) densities on top of representative geometries of the most important clusters of ds-DNA+**2**.

## Supplementary Material



## Notes

NMR and IR spectra, MS, X-ray, and electrochemical data for all compounds. Figures providing spectra stability studies, cell survival, the computational details of the simulations and a more detailed discussion of the analysis of the trajectories can be found in the Supporting Information (PDF)
