# Supplementary material for: Reimagining Pt(II) Anticancer Agents: The Role of Ferrocene in Monofunctional Chemotherapeutic Compounds
Source: Inorg Chem. 2025 Jun 2;64(23):11497–509. doi: 10.1021/acs.inorgchem.5c00704 (PMC12175133; doi:10.1021/acs.inorgchem.5c00704)
Supplement: Supplementary file 1 [file ic5c00704_si_001.pdf]

Reimagining Pt(II) Anticancer Agents: The Role of Ferrocene in Monofunctional Chemotherapeutic Compounds.

Selma Miguel,[1,2‡] Javier Ortín-Fernández,[1‡] Silvia Gómez-Pastor,[3] María Ángeles Moliné,[3] Pedro A. Sánchez-Murcia,[4,5] Inés Corral,[1,6*] Francisco Sanz-Rodríguez,[3*] Ana María González-Vadillo[2*]

[1]Departamento de Química, Facultad de Ciencias, Universidad Autónoma de Madrid, 28049 Madrid, Spain

[2]Departamento de Química Inorgánica, Facultad de Ciencias, Universidad Autónoma de Madrid, 28049 Madrid, Spain

[3]Departamento de Biología, Facultad de Ciencias, Universidad Autónoma de Madrid, 28049 Madrid, Spain

[4]Laboratory for Computer-Aided Molecular Design, Division of Medicinal Chemistry, Otto Loewi Research Center, Medical University Graz, Neue Stiftingtalstrasse 6/III, 8010- Graz, Austria

[5]BioTechMed-Graz, Mozartgasse 12/II 8010 Graz, Austria

[6]Institute for Advanced Research in Chemical Sciences (IAdChem), Universidad Autónoma de Madrid, 28049 Madrid, Spain

[‡]Equally contributed to this work

*corresponding authors: ines.corral@uam.es; francisco.sanz@uam.es; anam.gonzalez@uam.es

Content

1. **NMR, INFRARED AND MASS SPECTRA OF THE PREPARED COMPLEXES** 2
2. **CRYSTALLOGRAPHIC DATA AND X-RAY STRUCTURES** 14
3. **CONDUCTIVITY MEASUREMENTS** 16
4. **ELECTROCHEMICAL MEASUREMENTS** 16
5. **STABILITY STUDIES IN DMSO-d_6/H_2O** 20
6. **CELL CULTURE STUDIES** 22
7. **COMPUTATIONAL DETAILS** 25
8. **REFERENCES** 35

1. NMR, INFRARED AND MASS SPECTRA OF THE PREPARED COMPLEXES

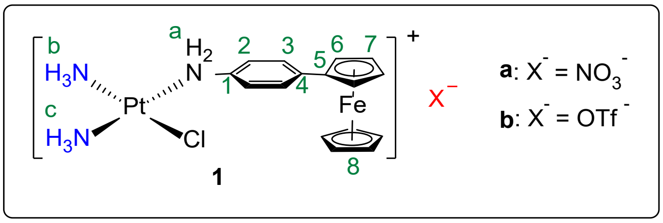

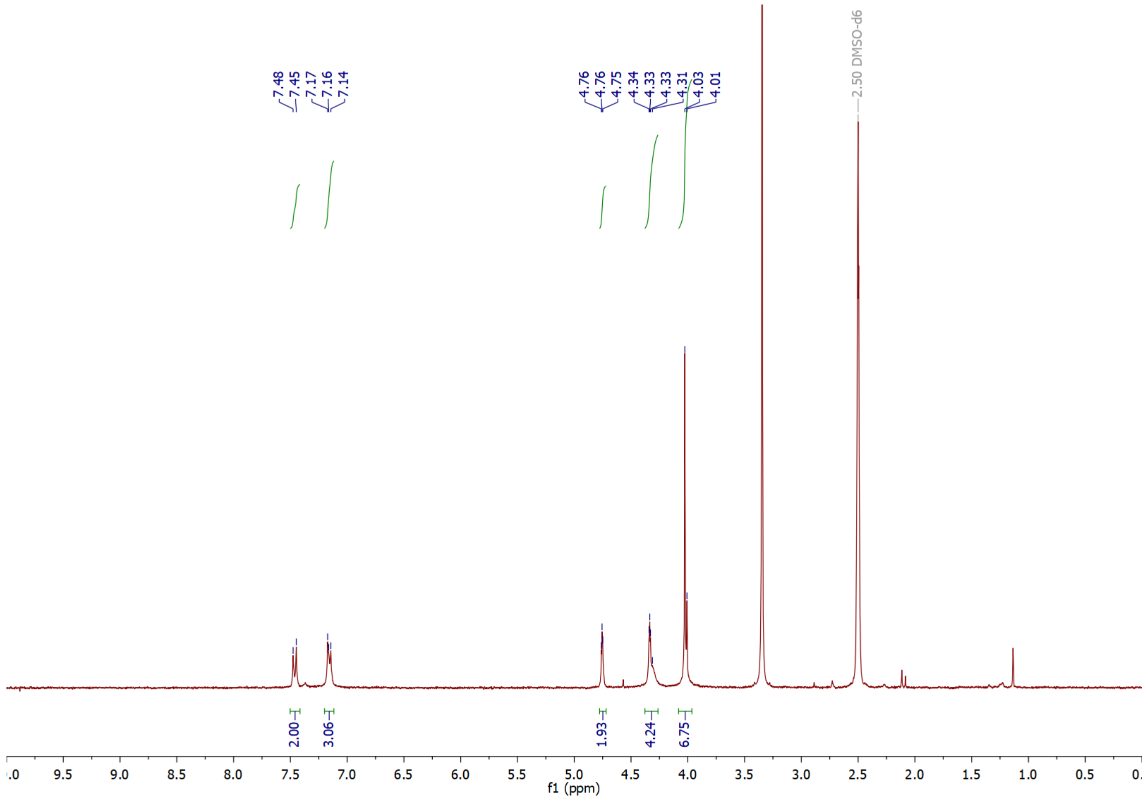

Figure S1. ^{1}H NMR spectra (300 MHz, DMSO-d6) of **1.a**.

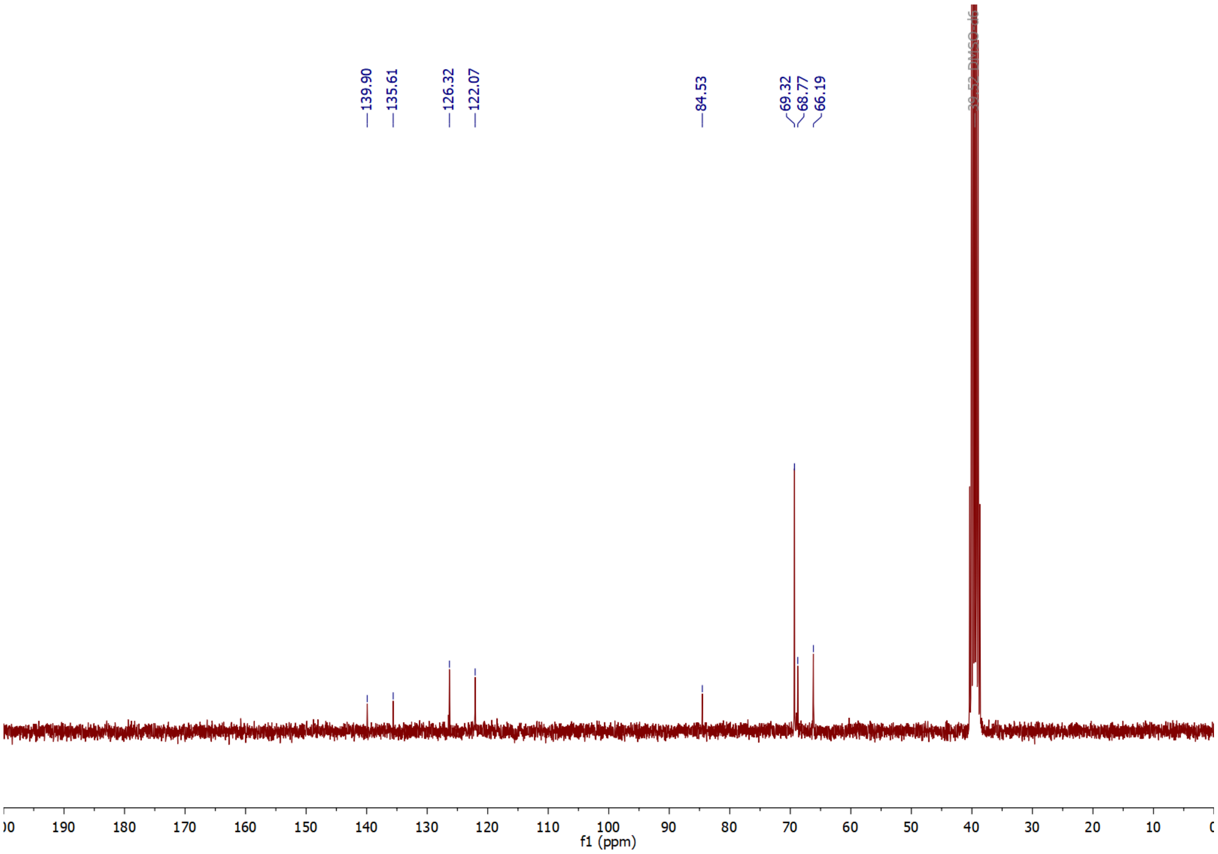

Figure S2. ^{13}C NMR spectra (75 MHz, DMSO-d_6) of **1.a**.

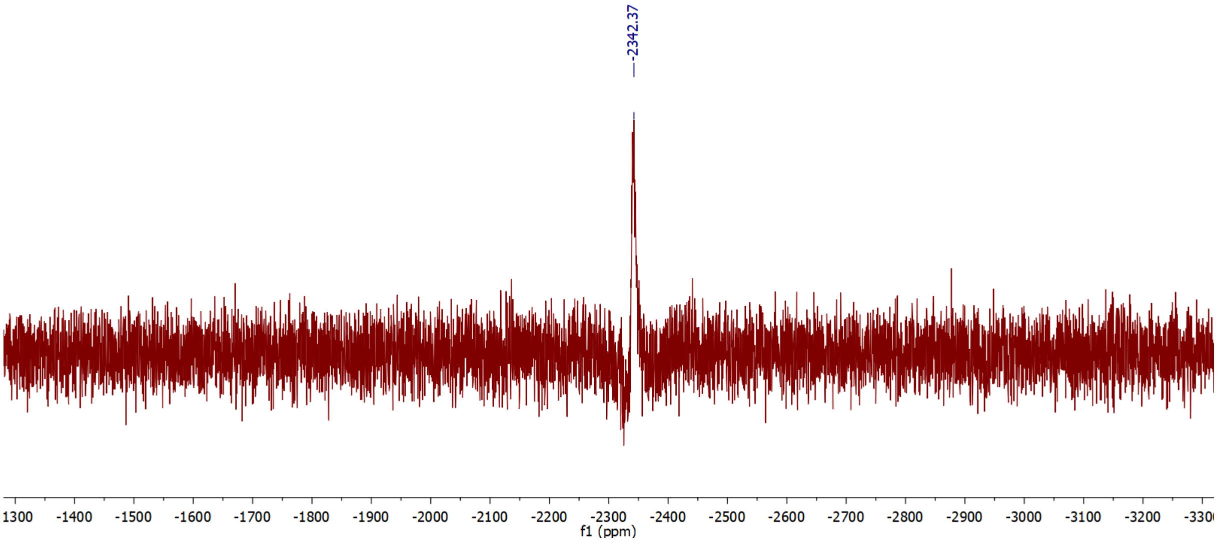

Figure S3. ^{195}Pt NMR spectra (64 MHz, DMSO-d_6) of **1.a**.

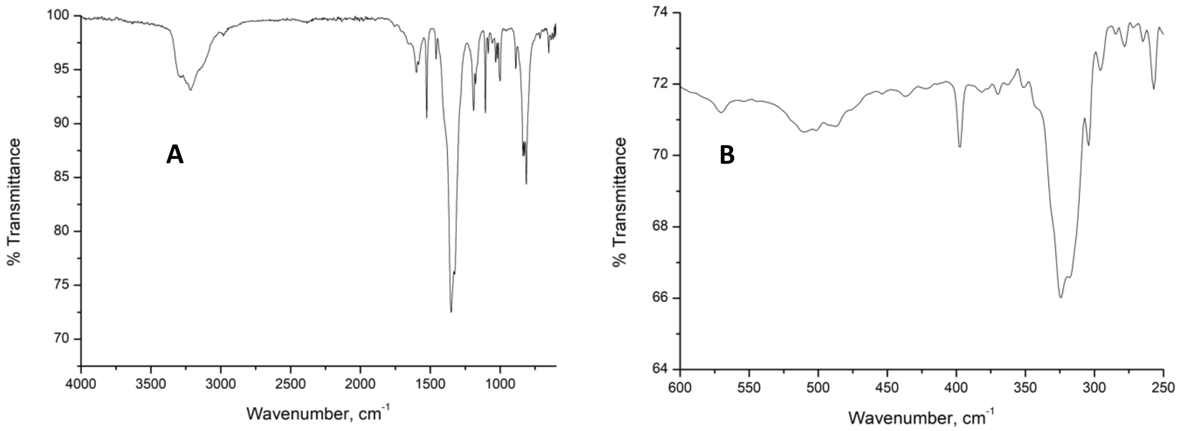

Figure S4. ATR (**A**) and Nujol suspension (**B**) infrared spectra of **1.a**.

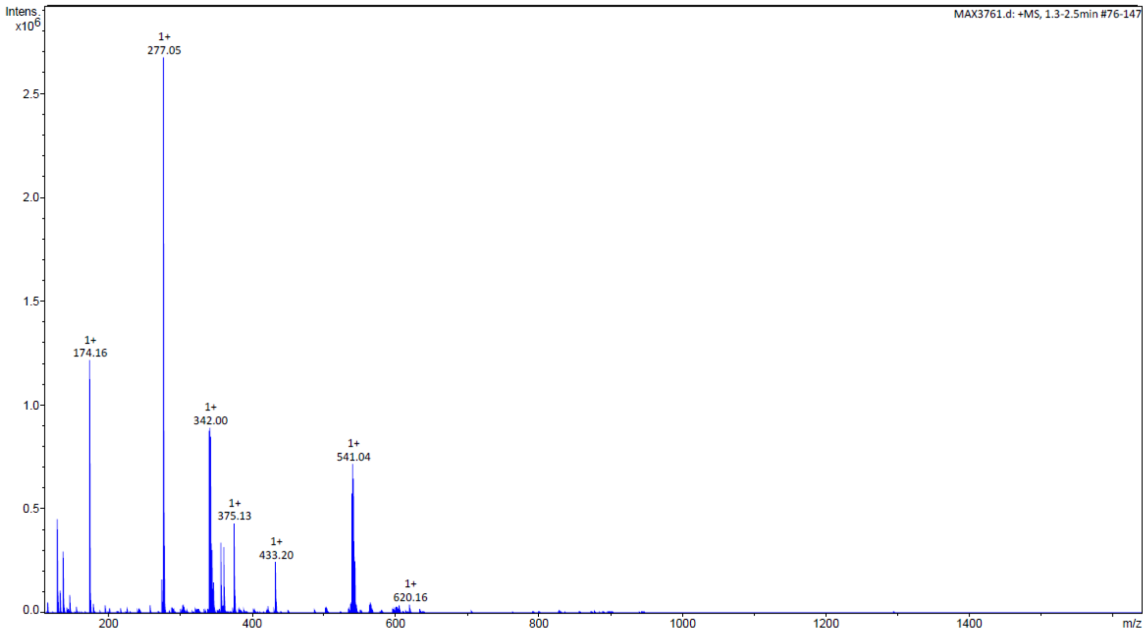

Figure S5. ESI$^+$ mass spectra of **1.a**.

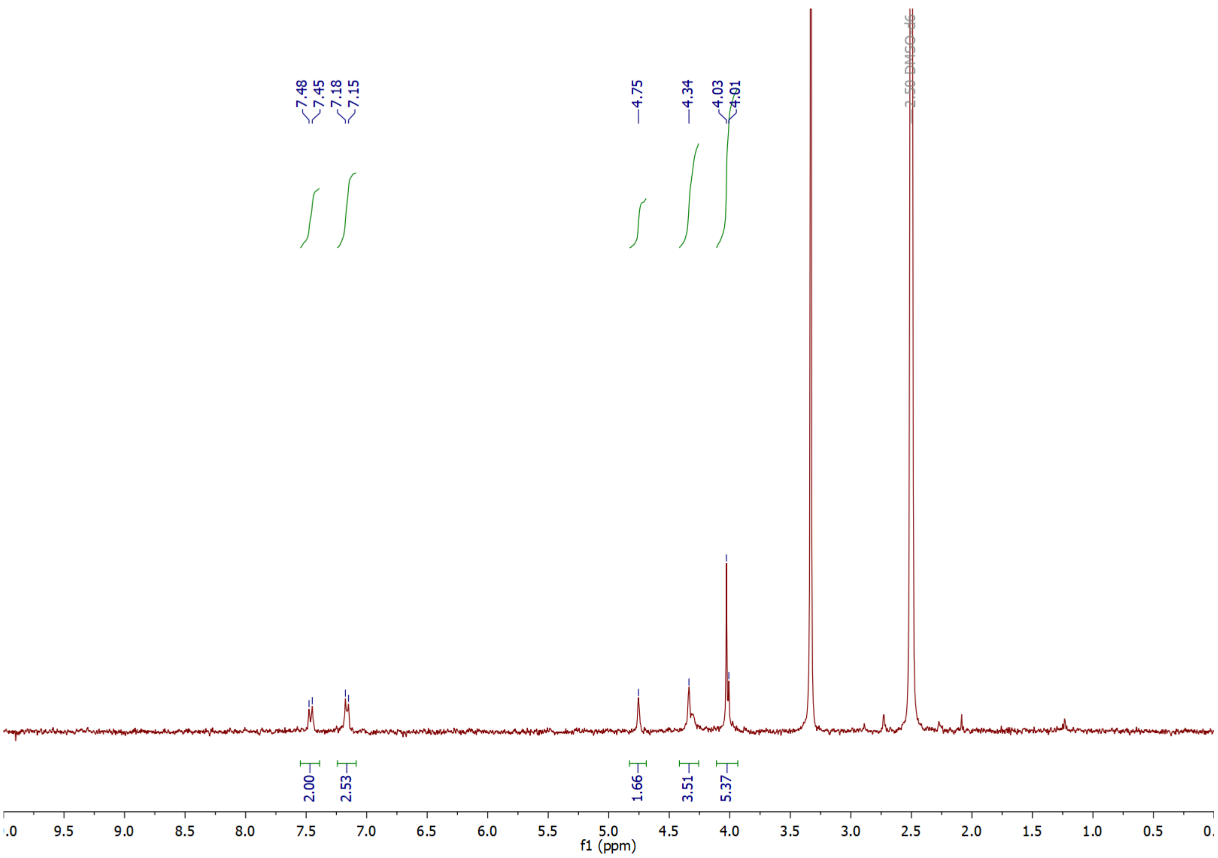

Figure S6. ^{1}H NMR spectra (300 MHz, DMSO-d_6) of **1.b**.

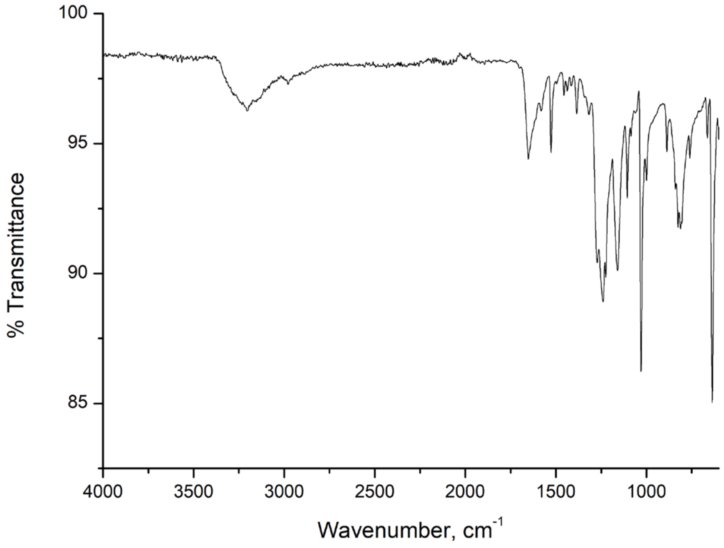

Figure S7. ATR infrared spectra of **1.b**.

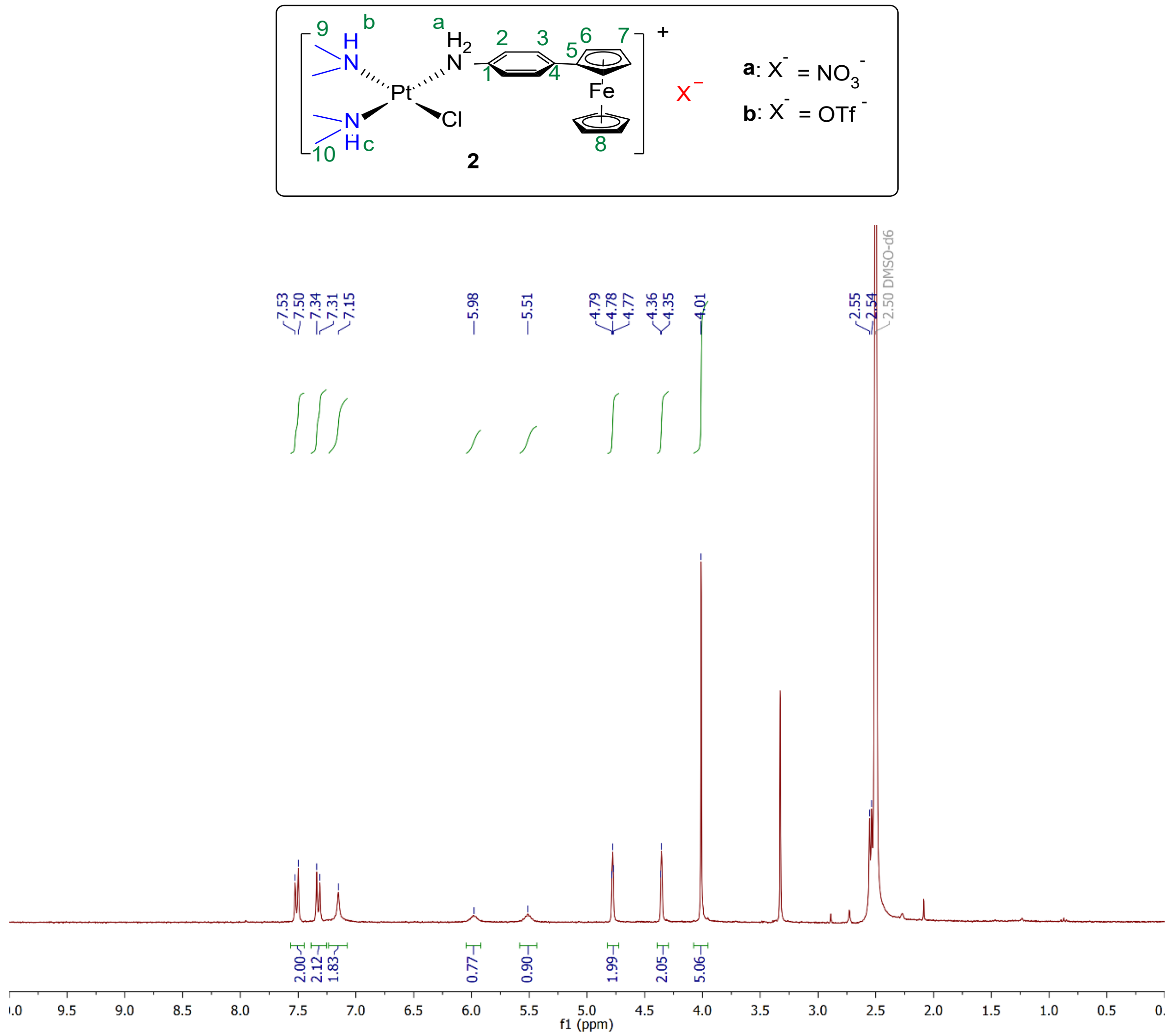

Figure S8. ^{1}H NMR spectra (300 MHz, DMSO-d_6) of **2.a**.

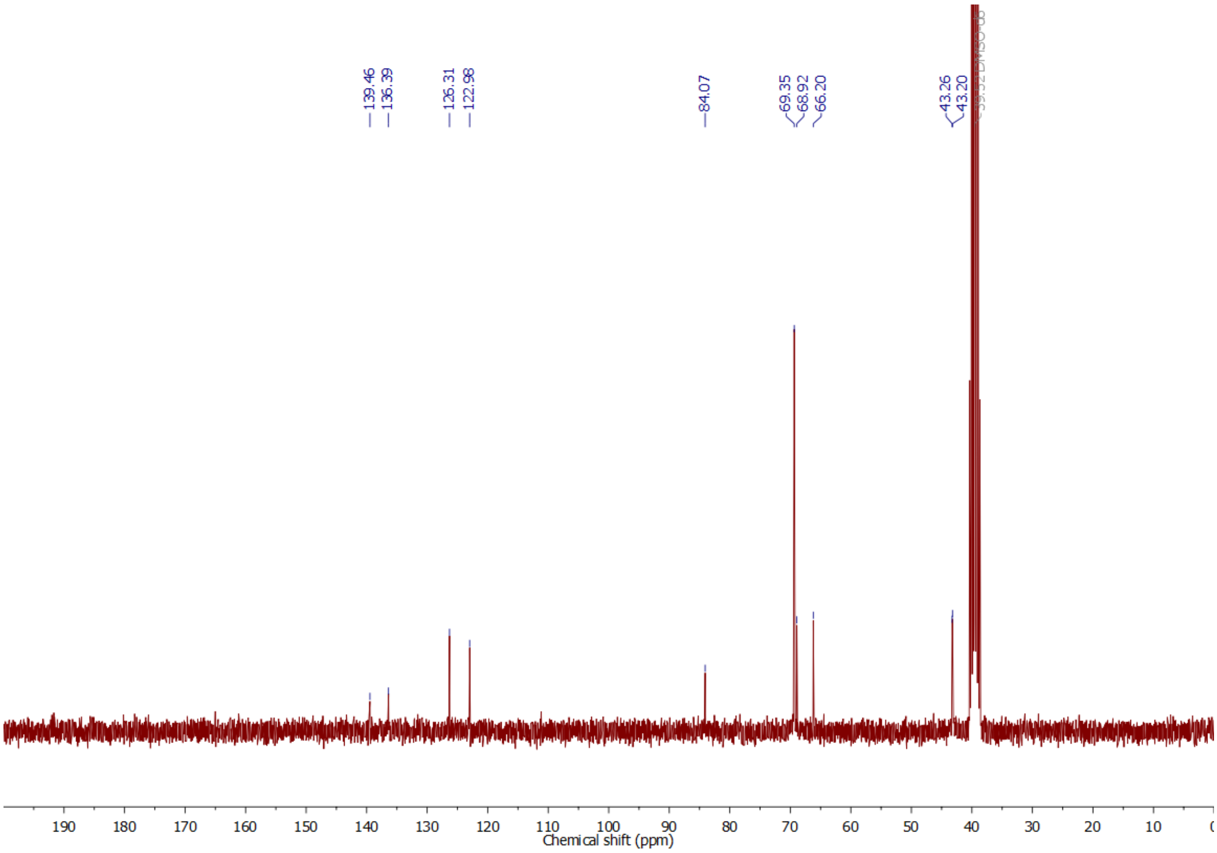

Figure S9. ^{13}C NMR spectra (75 MHz, DMSO-d$_6$) of **2.a**.

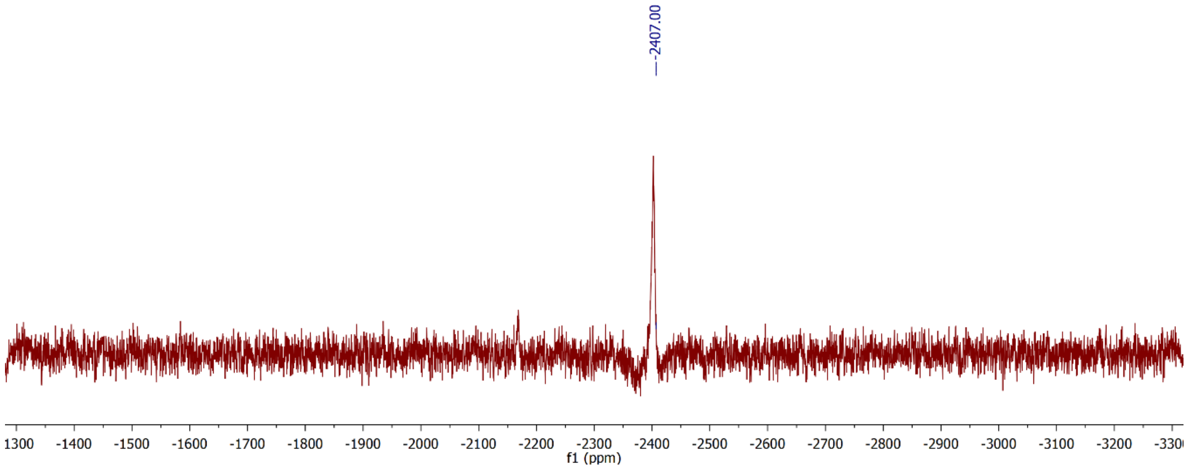

Figure S10. ^{195}Pt NMR spectra (64 MHz, DMSO-d$_6$) of **2.a**.

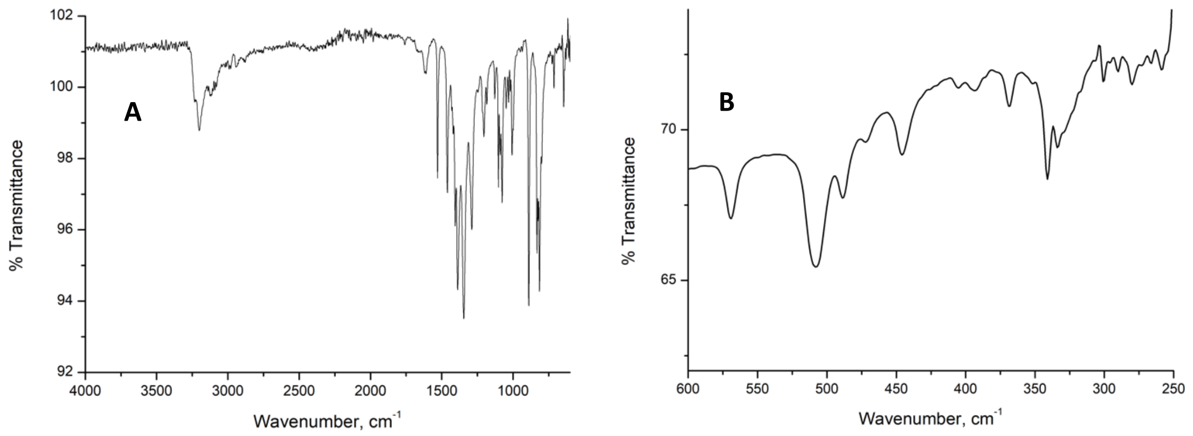

Figure S11. ATR (**A**) and Nujol suspension (**B**) infrared spectra of **2.a**.

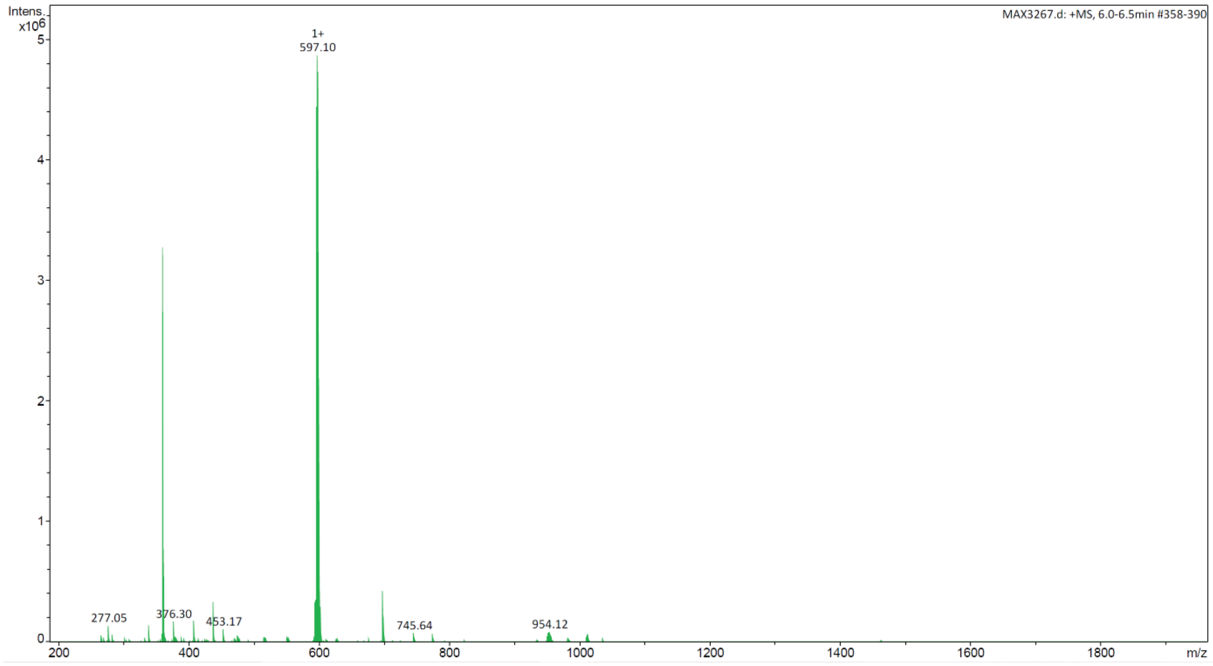

Figure S12. ESI+ mass spectra of **2.a**.

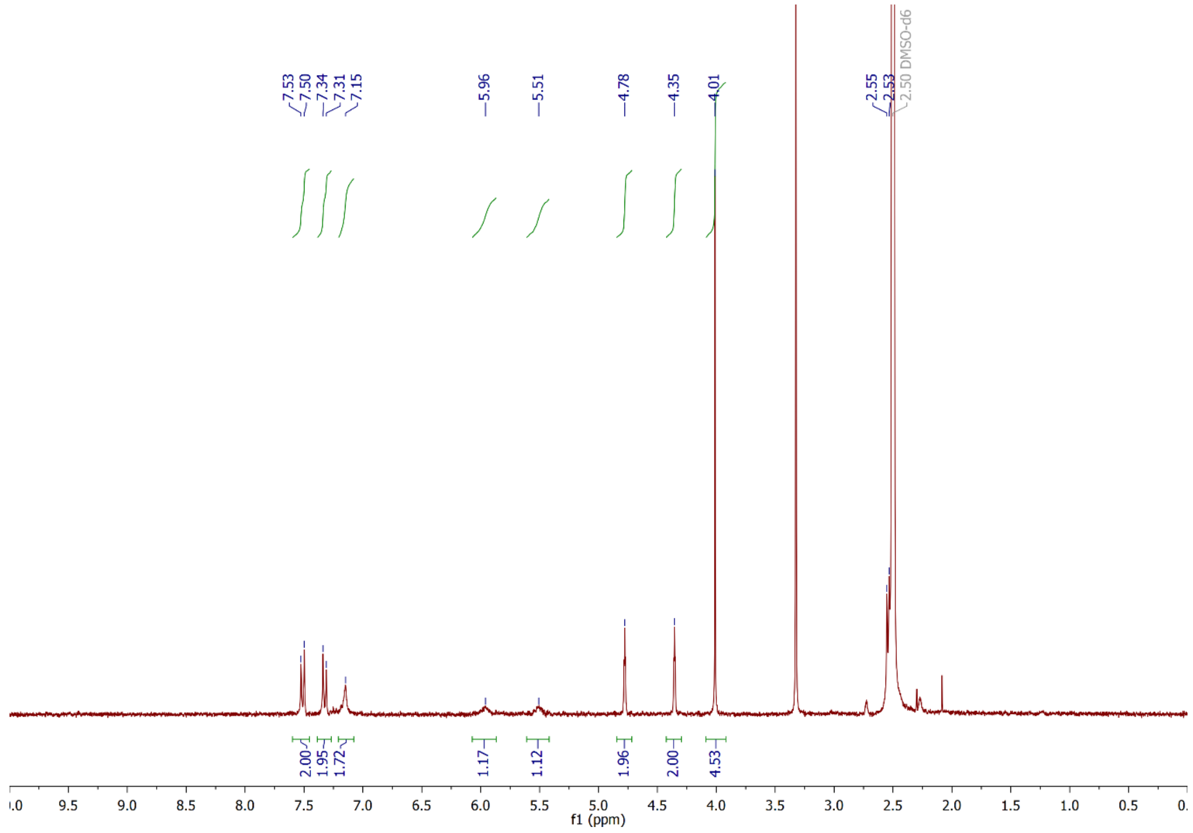

Figure S13. ^{1}H NMR spectra (300 MHz, DMSO-d$_6$) of **2.b**.

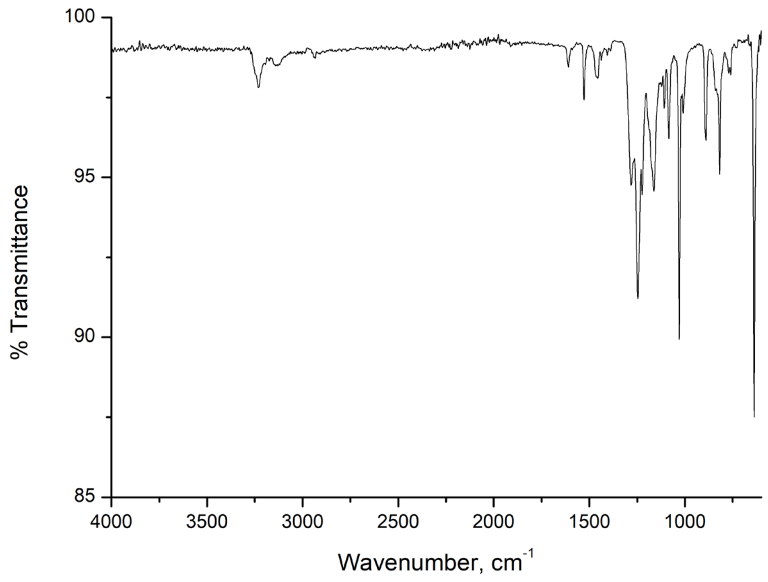

Figure S14. ATR infrared spectra of **2.b**.

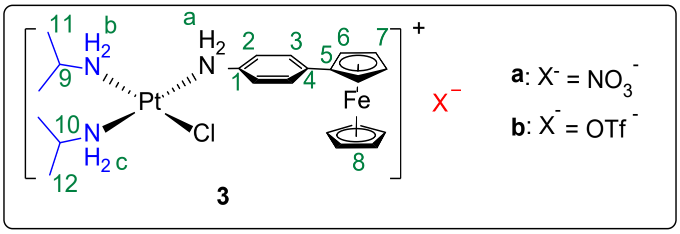

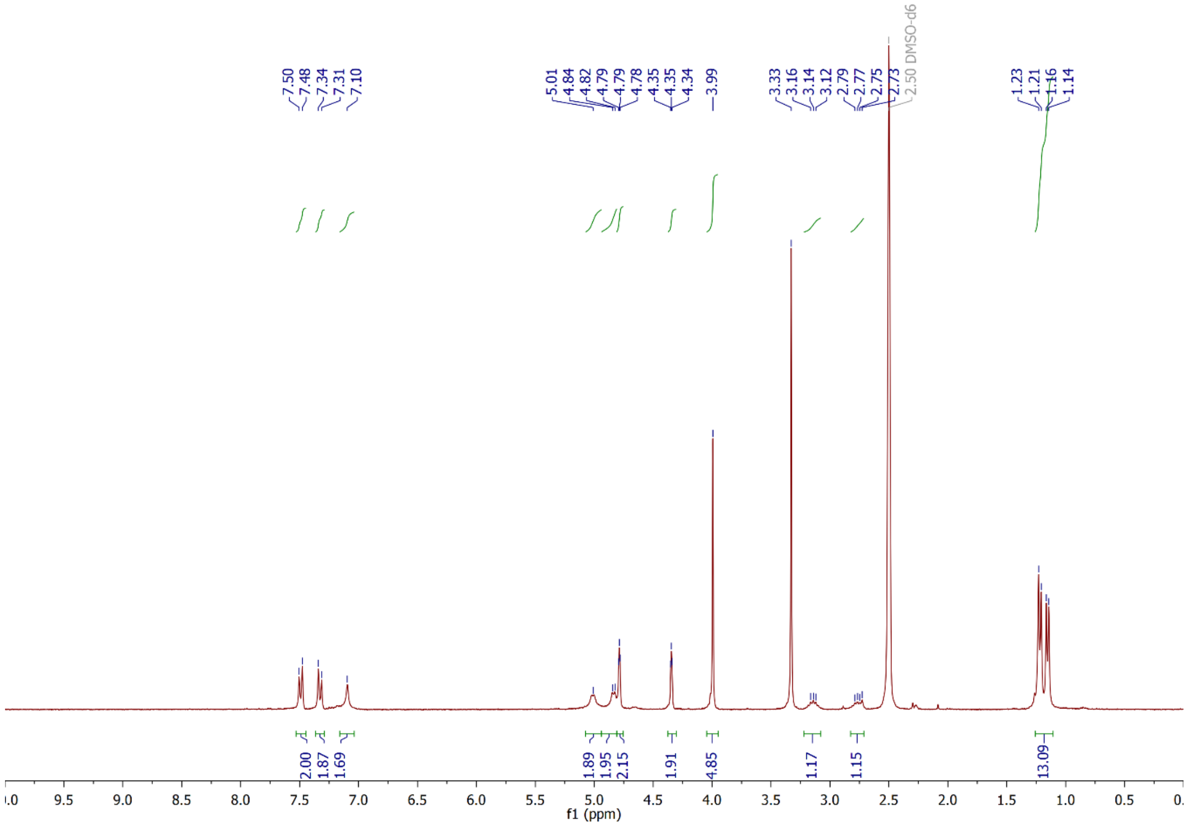

Figure S15. ^{1}H NMR spectra (300 MHz, DMSO-d$_6$) of **3.a**.

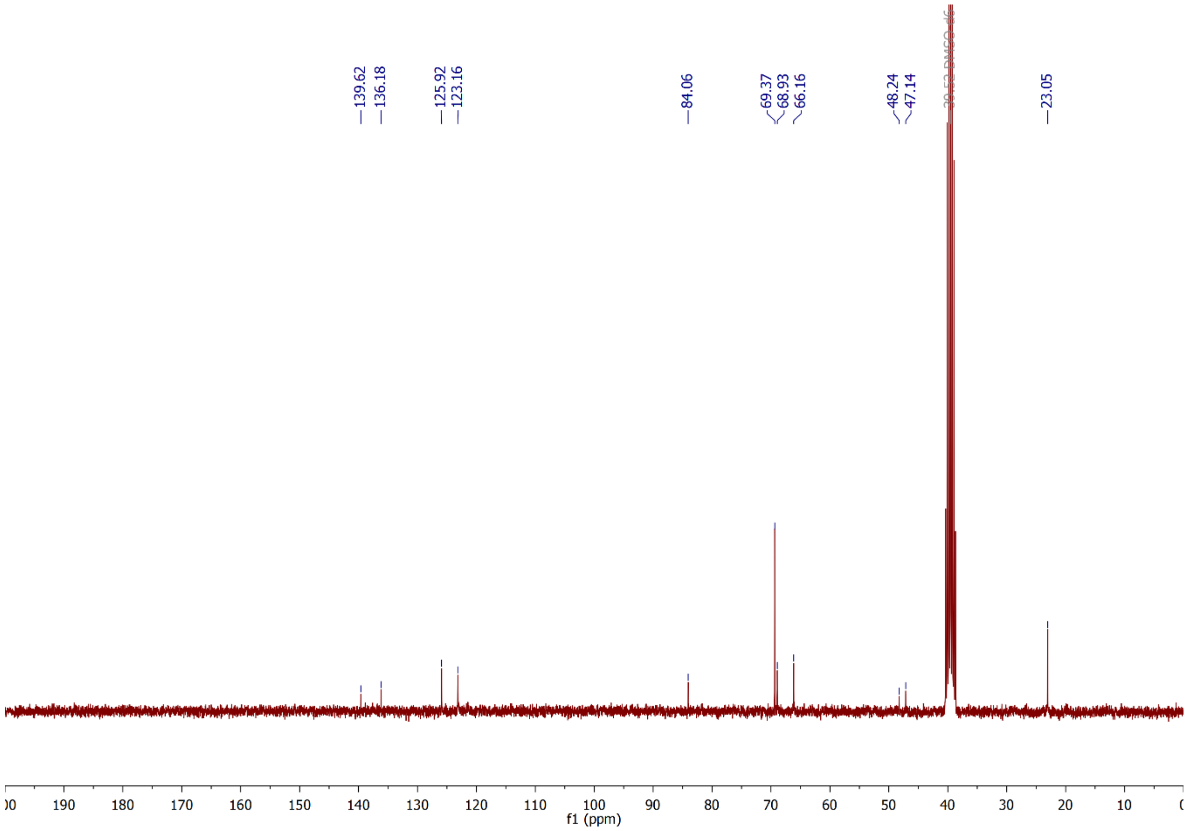

Figure S16. ^{13}C NMR spectra (75 MHz, DMSO-d_6) of **3.a**.

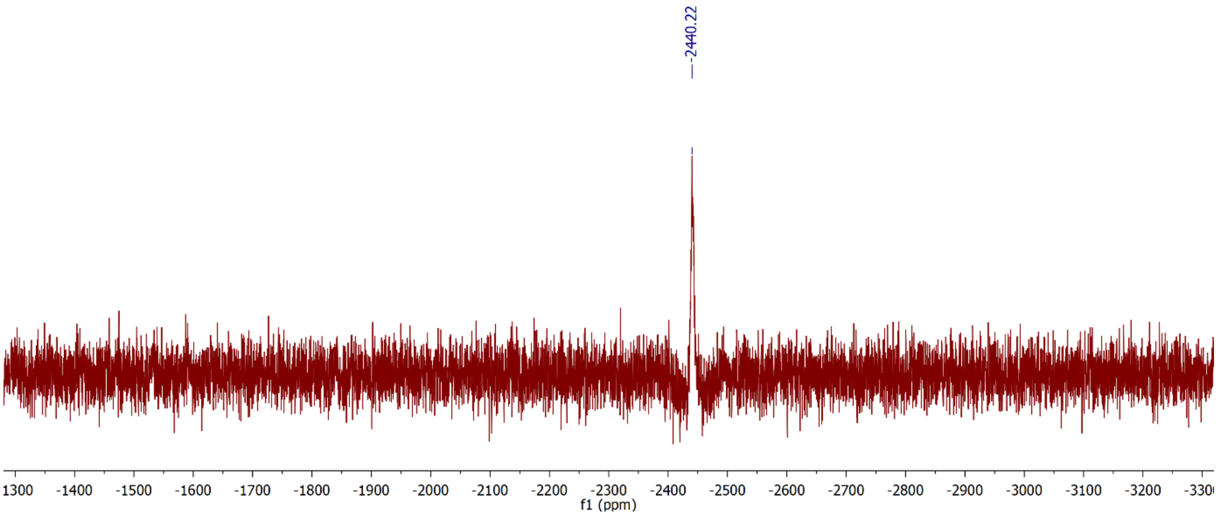

Figure S17. ^{195}Pt NMR spectra (64 MHz, DMSO-d_6) of **3.a**.

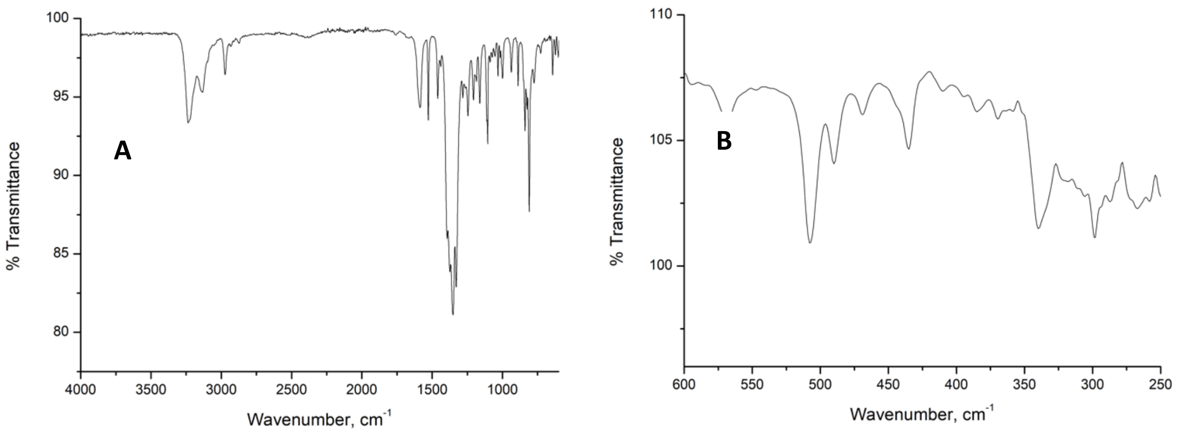

Figure S18. ATR (**A**) and Nujol suspension (**B**) infrared spectra of **3.a**.

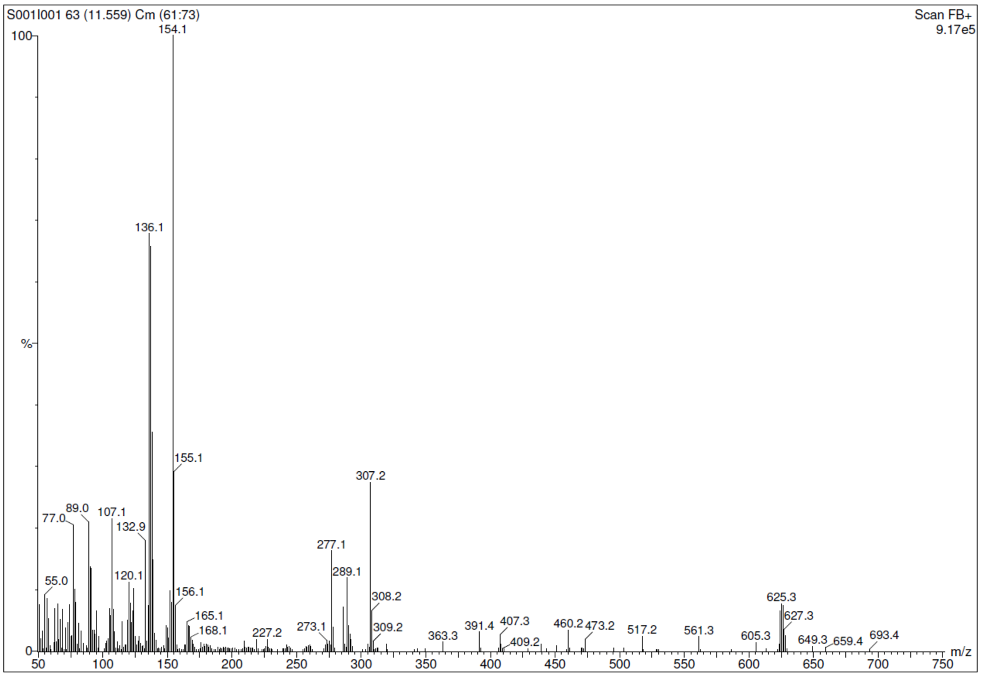

Figure S19. FAB mass spectra of **3.a**.

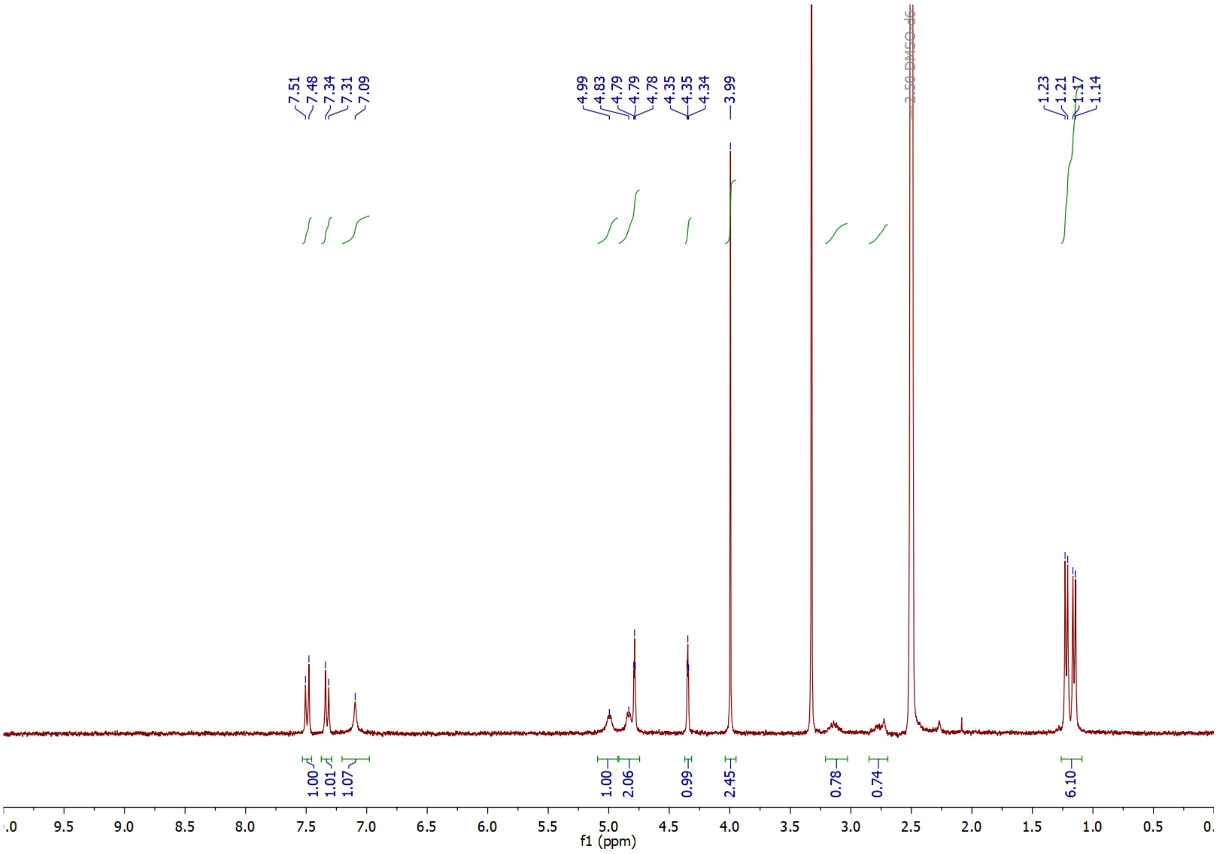

Figure S20. ¹H NMR spectra (300 MHz, DMSO-d_6) of **3.b**.

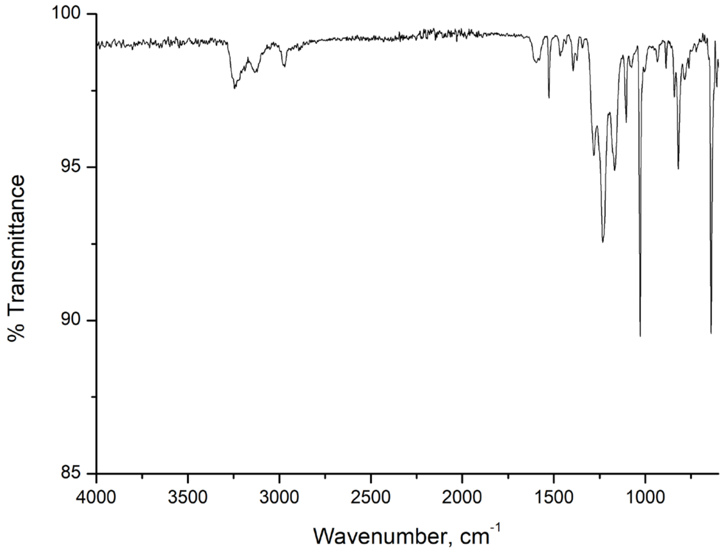

Figure S21. ATR infrared spectra of **3.b**.

2. CRYSTALLOGRAPHIC DATA AND X-RAY STRUCTURES

Table S1. Crystal data and structure refinement for complexes **2.b** and **3.b**.

Compound	**2.b**	**3.b**
Empirical formula	$C_{21}H_{29}ClF_3FeN_3O_3PtS$	$C_{23}H_{33}ClF_3FeN_3O_3PtS$
Fw	746.92	774.97
T [K]	296(2)	250(2)
λ [Å]	0.71073	0.71073
cryst syst	Monoclinic	Orthorhombic
Space group	$P2_1/n$ 1	$P\,2_1\,2_1\,2_1$
a, Å	11.4489(3)	6.0917(2)
b, Å	6.8547(2)	16.7615(7)
α, deg	90	90
β, deg	94.5180(10)	90
c, Å	32.9972(7)	32.2165(12)
γ, deg	90	90
V, Å^3	2581.53(12)	3289.5(2)
Z	4	4
Density (calcd), mgm^{-3}	1.922	1.565
μ, mm^{-1}	6.209	4.876
F(000)	1456	1520
Crystal size, mm^3	0.033 x 0.078 x 0.127	0.023 x 0.036 x 0.250
θ, deg	1.93 to 25.35	1.75 to 25.35
Index ranges	$-13 \leq h \leq 13$<br>$-8 \leq k \leq 8$<br>$-39 \leq l \leq 39$	$-7 \leq h \leq 7$<br>$-20 \leq k \leq 20$<br>$-38 \leq l \leq 38$
no. of reflns collected	27135	42169
no. of indep rflns	4718 [R(int) = 0.0283]	6025 [R(int) = 0.0617]
completeness	99.7% (to θ = 25.35°)	99.7% (to θ = 25.35°)
absorp corr	Multi-Scan	Multi-Scan
refinement method	Full-matrix least-squares on $F2$	Full-matrix least-squares on $F2$
no. of data/restraints/params	4718/0/299	6025/417/385
goodness-of-fit on $F2$	1.080	1.015
Final R indices ($I > 2\sigma(I)$)	$R_1 = 0.0422$, w$R_2 = 0.1031$	$R_1 = 0.0360$, w$R_2 = 0.0861$
R indices (all data)	$R_1 = 0.0509$, w$R_2 = 0.1082$	$R_1 = 0.0470$, w$R_2 = 0.0910$
largest diff peak and hole/e Å^{-3}	1.774 and -1.936	0.918 and -1.068

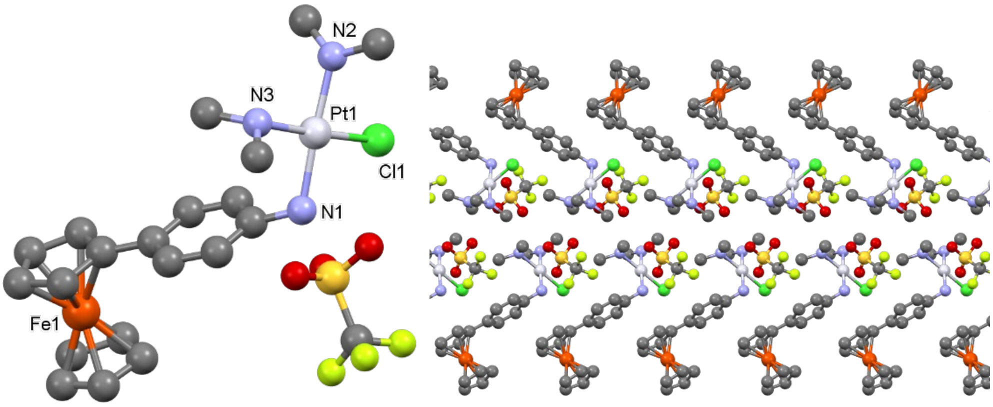

Figure S22. Molecular structure of **2.b** (left, CCDC 2361678), hydrogen atoms have been omitted for clarity. Crystal-packing diagram of complex **2.b** along the *a*-axis (right).

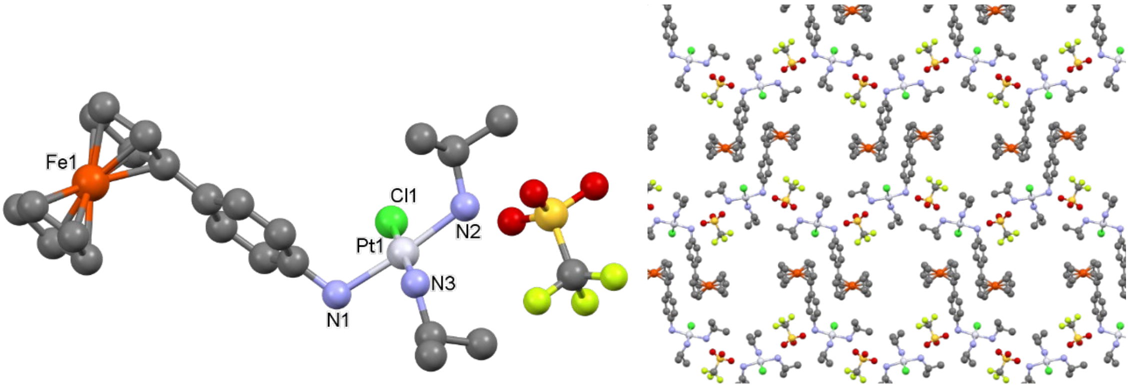

Figure S23. Molecular structure of **3.b** (left, CCDC 2361679), hydrogen atoms have been omitted for clarity. Crystal-packing diagram of complex **3.b** along the *a*-axis (right). Only fluorine and oxygen atoms in position A are shown.

Table S2. Selected bond lengths (Å) and angles (°) for compound **2.b**.

Pt(1)–Cl(1)	2.284(3)	N(1)–Pt(1)–N(2)	176.6(4)
Pt(1)–N(1)	2.062(8)	N(3)–Pt(1)–Cl(1)	177.3(2)
Pt(1)–N(2)	2.053(8)	N(1)–Pt(1)–Cl(1)	86.1(3)
Pt(1)–N(3)	2.062(9)	Cl(1)–Pt(1)–N(2)	90.7(3)
Pt(1)–Fe(1)	8.483	N(2)–Pt(1)–N(3)	87.3(4)
		N(3)–Pt(1)–N(1)	95.9(4)

Table S3. Selected bond lengths (Å) and angles (°) for compound **3.b**.

Pt(1)–Cl(1)	2.288(3)	N(1)–Pt(1)–N(2)	174.9(3)
Pt(1)–N(1)	2.083(8)	N(3)–Pt(1)–Cl(1)	177.4(2)
Pt(1)–N(2)	2.053(9)	N(1)–Pt(1)–Cl(1)	88.4(3)
Pt(1)–N(3)	2.048(8)	Cl(1)–Pt(1)–N(2)	88.4(3)
Pt(1)–Fe(1)	8.470	N(2)–Pt(1)–N(3)	91.6(4)
		N(3)–Pt(1)–N(1)	91.8(4)

3. CONDUCTIVITY MEASUREMENTS

Table S4. Conductivity of the heterometallic complexes **1.a** – **3.b** (S cm^2 mol^{-1}).

**1.a**	57.0	**1.b**	52.2
**2.a**	50.8	**2.b**	50.5
**3.a**	56.0	**3.b**	50.0

4. ELECTROCHEMICAL MEASUREMENTS

Table S5. Electrochemical data of the prepared compounds at a scan rate of 0.100 V s^{-1}.

	$\|i_c/i_a\|$	ΔE_p (V vs SCE)	$E_{1/2}$ CV (V vs SCE)	$E_{1/2}$ SWV (V vs SCE)
**L**	0.9	0.074	0.380	0.383
**1.b**	1.0	0.073	0.509	0.513
**2.b**	1.0	0.075	0.508	0.503
**3.b**	1.0	0.074	0.514	0.512

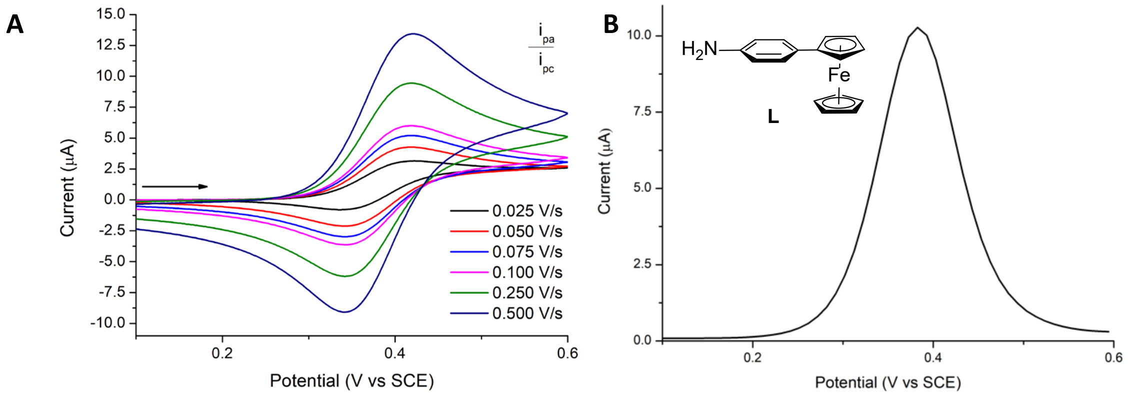

Figure S24. Cyclic voltammetries at different scan rates (**A**) and SWV (**B**) of the ligand **L** (10^{-3} M) in acetone 0.2 M n-Bu_4NPF_6.

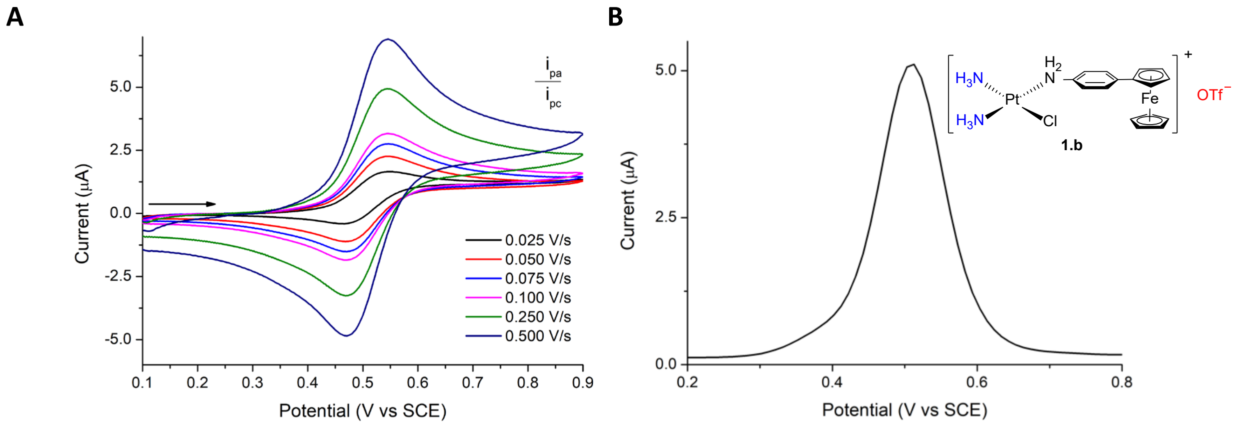

Figure S25. Cyclic voltammetries at different scan rates (**A**) and SWV (**B**) of complex **1.b** (10^{-3} M) in acetone 0.2 M n-Bu_4NPF_6.

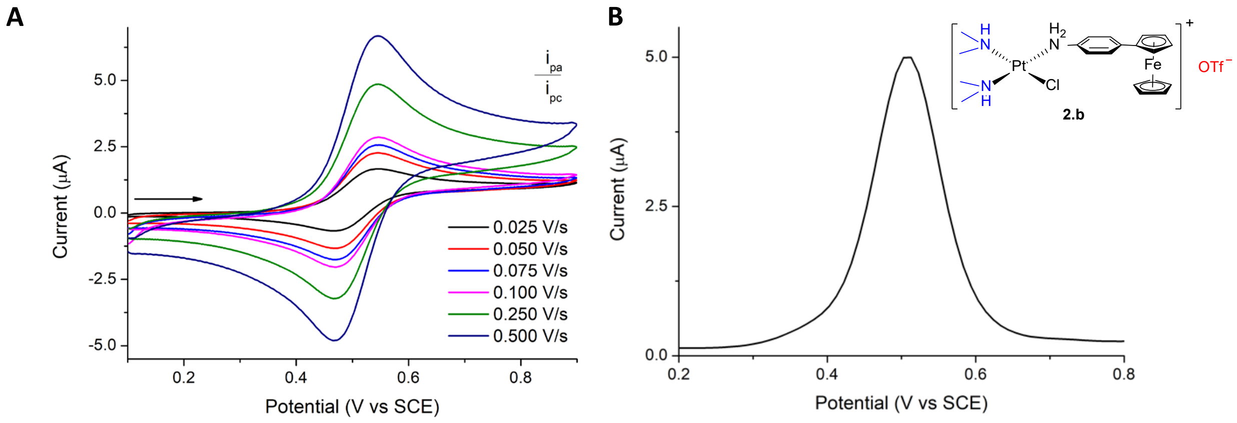

Figure S26. Cyclic voltammetries at different scan rates (**A**) and SWV (**B**) of complex **2.b** (10^{-3} M) in acetone 0.2 M n-Bu_4NPF_6.

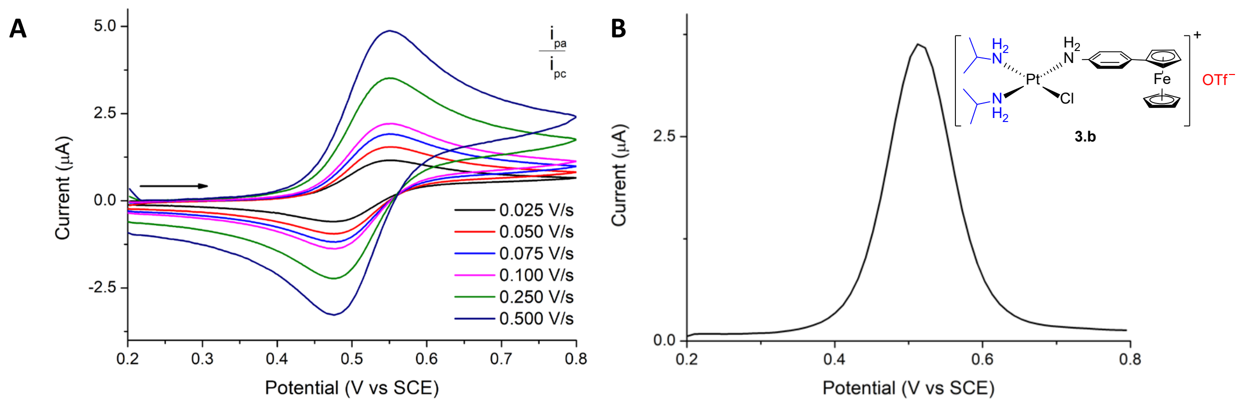

Figure S27. Cyclic voltammetries at different scan rates (**A**) and SWV (**B**) of complex **3.b** (10^{-3} M) in acetone 0.2 M n-Bu_4NPF_6.

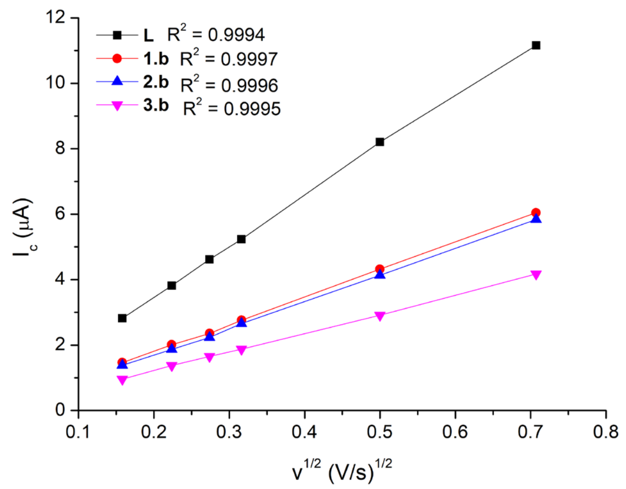

Figure S28. Representation of the Randles-Sevcik equation for **L** and the heterometallic complexes **1.b**-**3.b**.

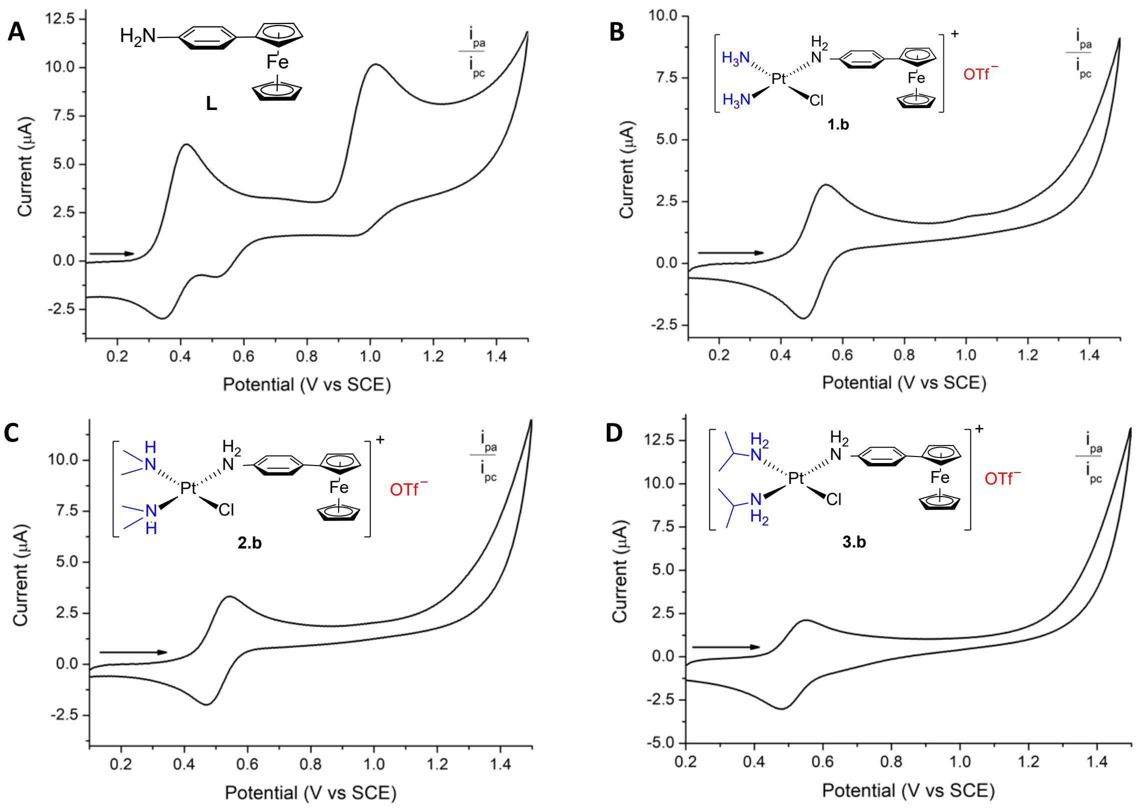

Figure S29. Cyclic voltammetries of **L** (**A**), **1.b** (**B**), **2.b** (**C**) and **3.b** (**D**) at 0.1 V/s in acetone increasing the anodic limit to 1.5 V.

5. STABILITY STUDIES IN DMSO-d$_6$/H_2O

^{1}H NMR spectra of 10^{-3} M solutions of the complexes **1.a**, **2.a** and **3.a** in DMSO-d$_6$/H_2O were recorded at different times (0h, 24h and 48h).

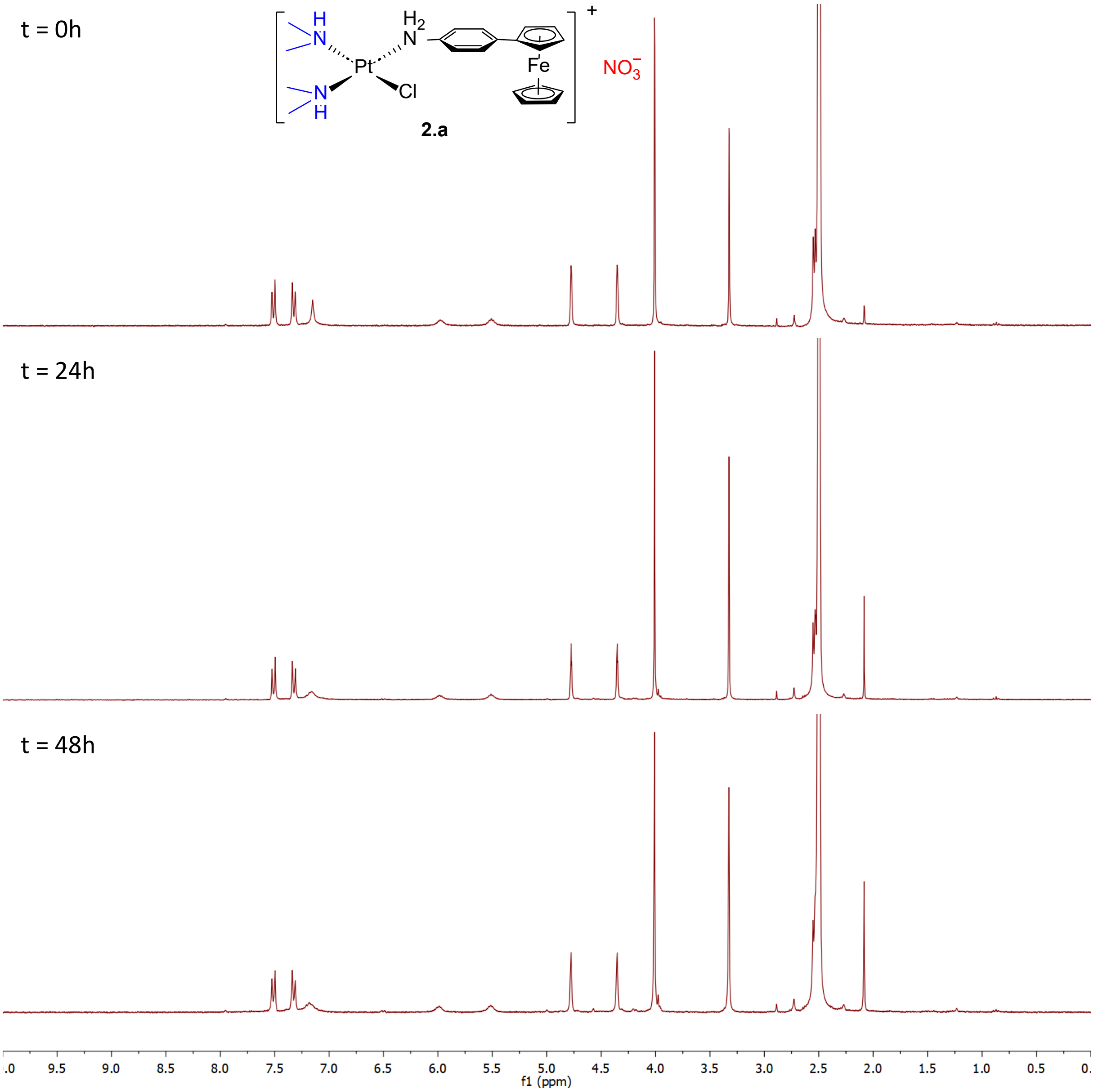

Figure S30. ^{1}H NMR spectra (300 MHz, DMSO-d$_6$) of **2.a** at different times.

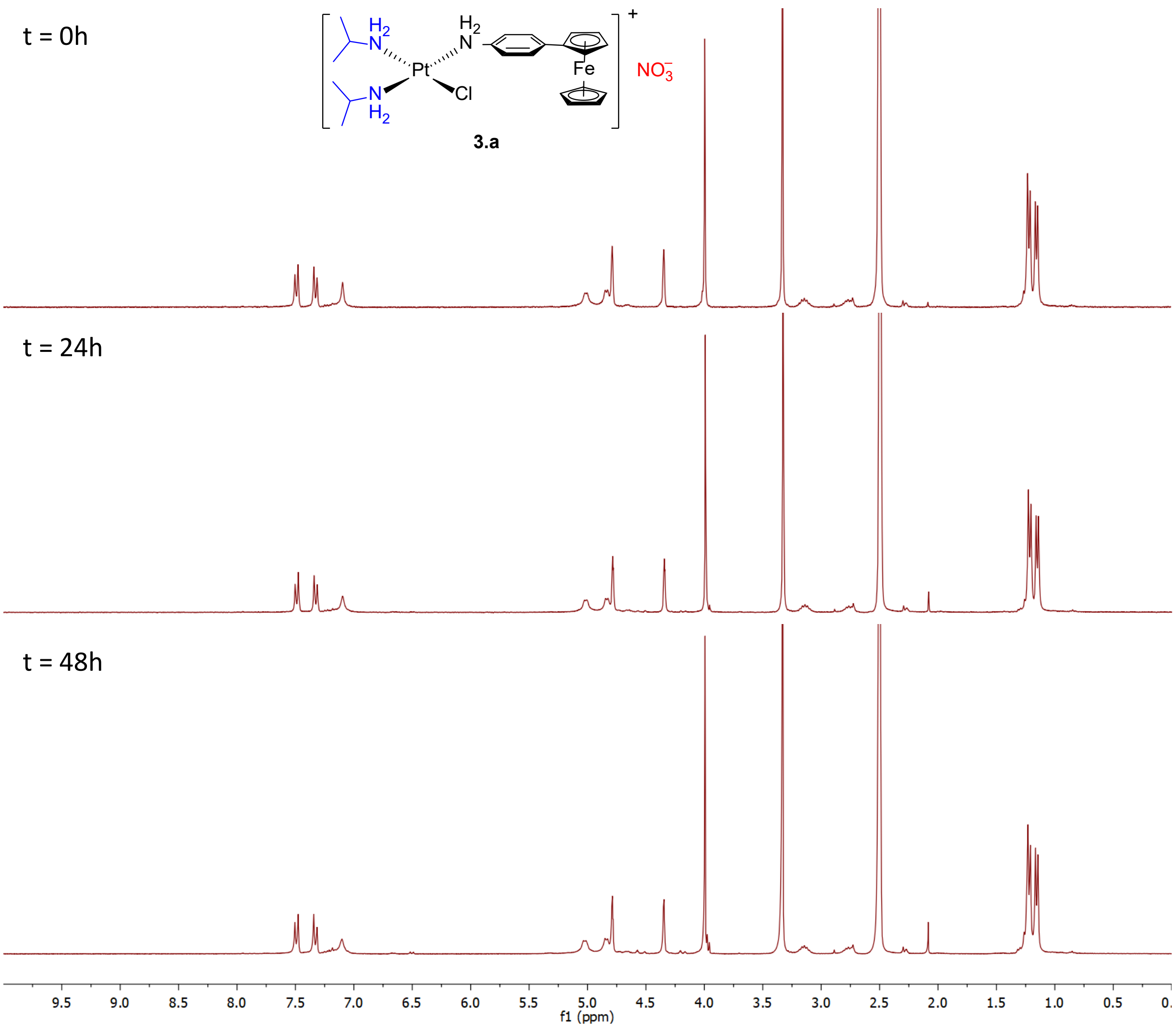

Figure S31. ^{1}H NMR spectra (300 MHz, DMSO-d_6) of **3.a** at different times.

6. CELL CULTURE STUDIES

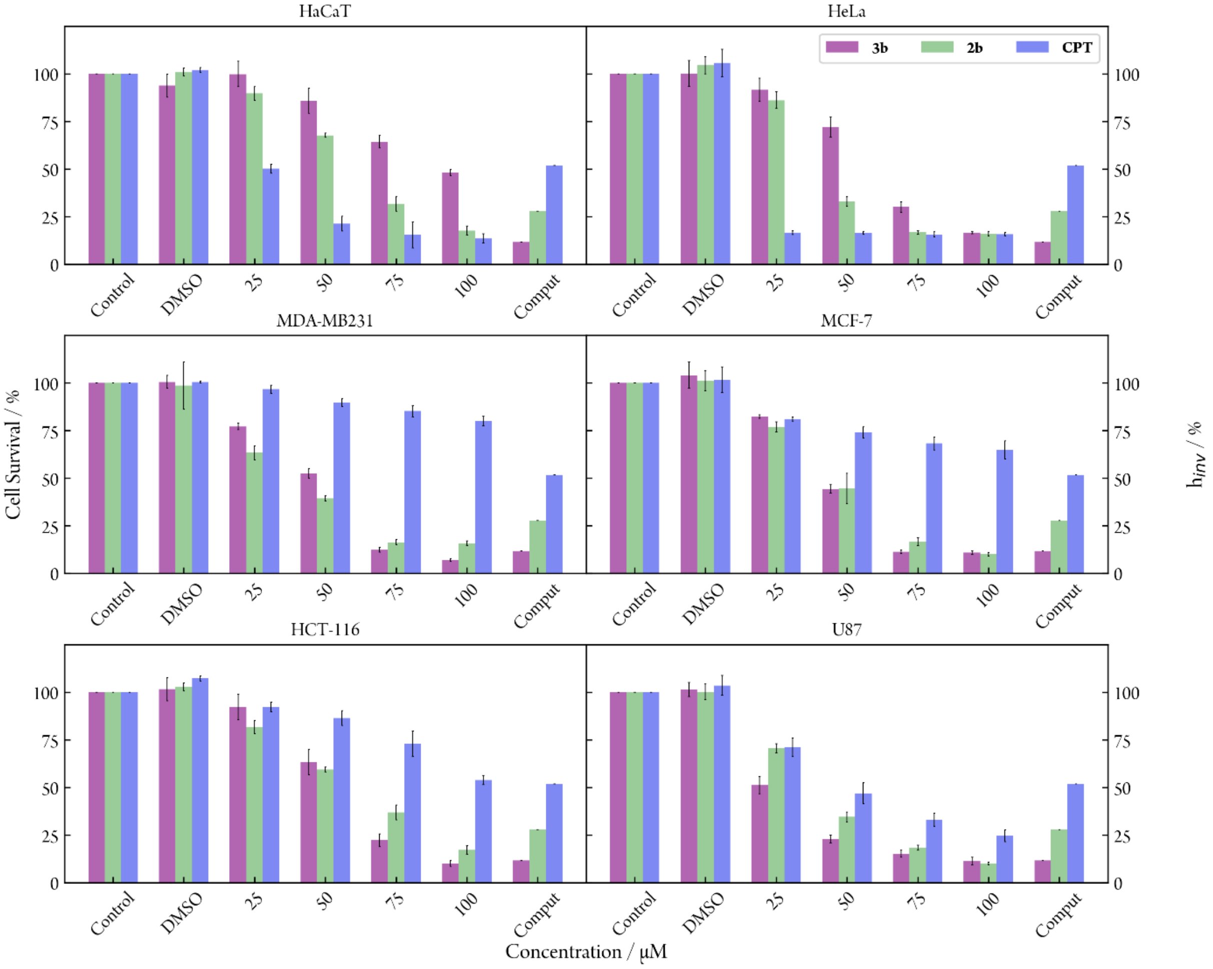

Figure S32. Cell survival of different tumoral and non-tumoral (HaCaT) cell lines upon exposure to selected concentrations of the nitrate salts of the complexes **2.b** (green), **3.b** (purple) or **CPT** (blue). The last group of bars in each panel refers to the duplex distortion measured as h_{inv} as obtained from computational simulations (see below for more details).

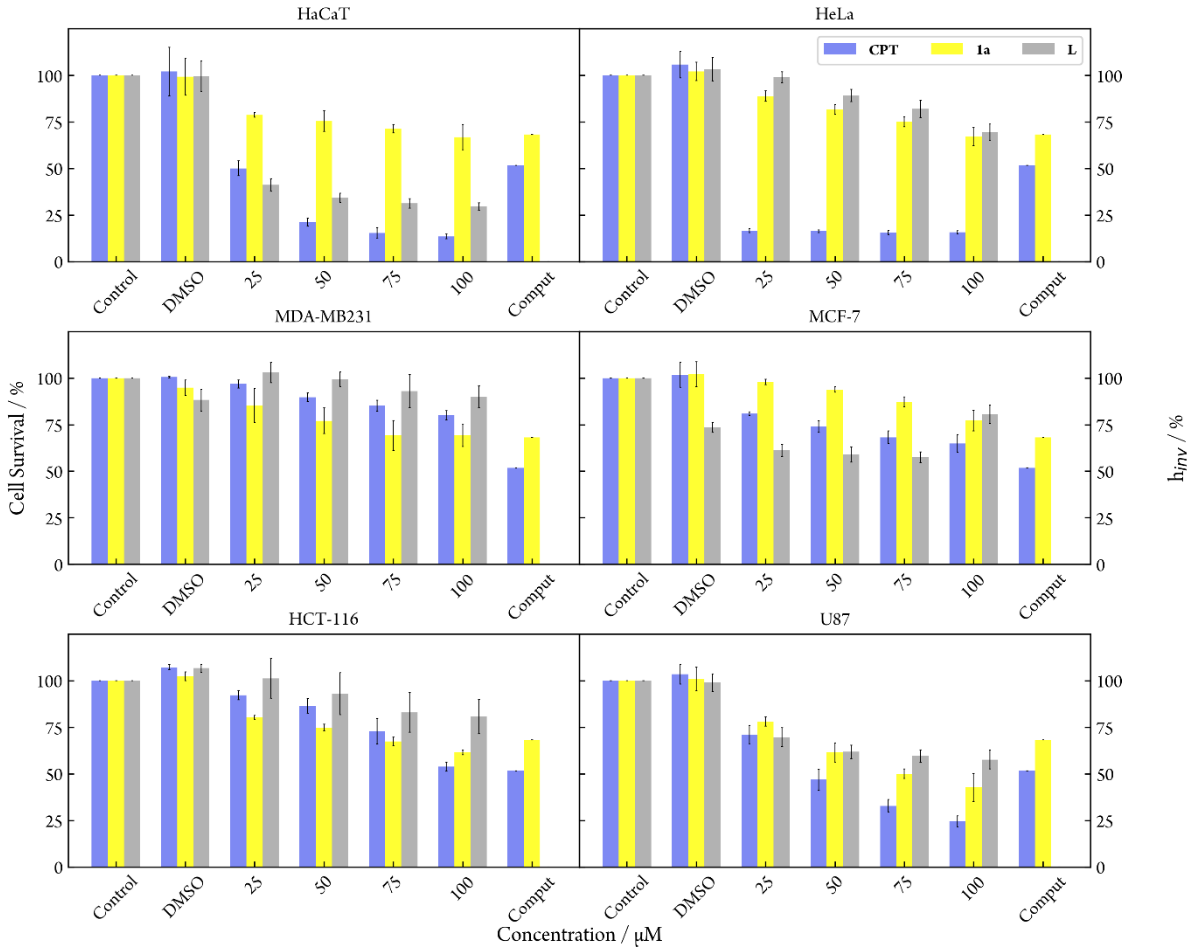

Figure S33. Cell survival of different tumoral and non-tumoral (HaCaT) cell lines upon exposure to selected concentrations of the nitrate salts of the complexes **1.a** (yellow), **L** (grey) or **CPT** (blue). The last group of bars in each panel refers to the duplex distortion measured as h_{inv} as obtained from computational simulations (see below for more details).

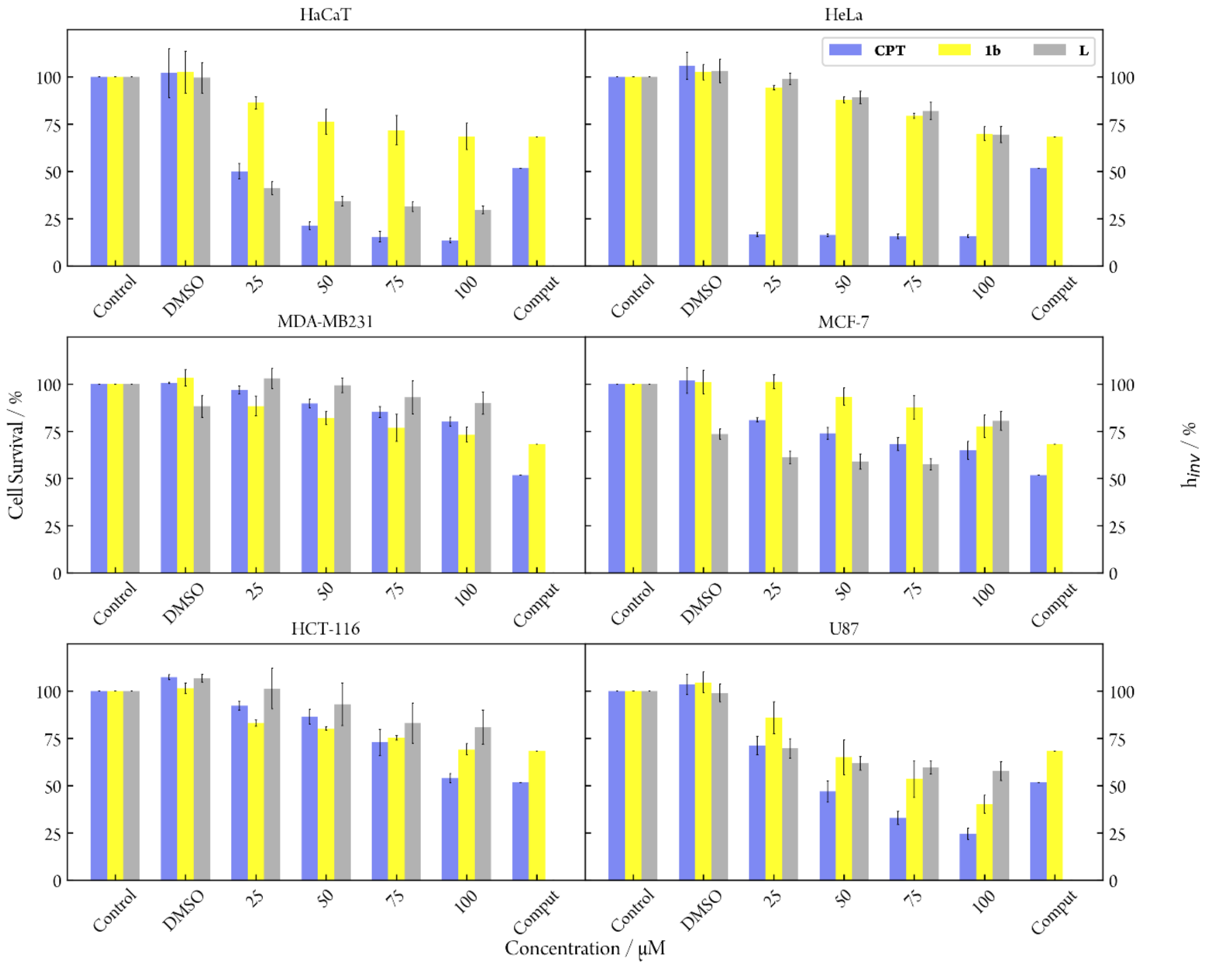

Figure S34. Cell survival of different tumoral and non-tumoral (HaCaT) cell lines upon exposure to selected concentrations of the nitrate salts of the complexes **1.b** (yellow), **L** (grey) or **CPT** (blue). The last group of bars in each panel refers to the duplex distortion measured as h_{inv} as obtained from computational simulations (see below for more details).

7. COMPUTATIONAL DETAILS

The initial DNA duplex structure with reference code 3LPV was extracted from the PDB[1]. This structure belongs to a crystallized intrastrand-platinated DNA double strand (ds-DNA), with the sequence 5'-CCTCTGGTCTCC-3', and the cisplatin bonded to the two central guanines.

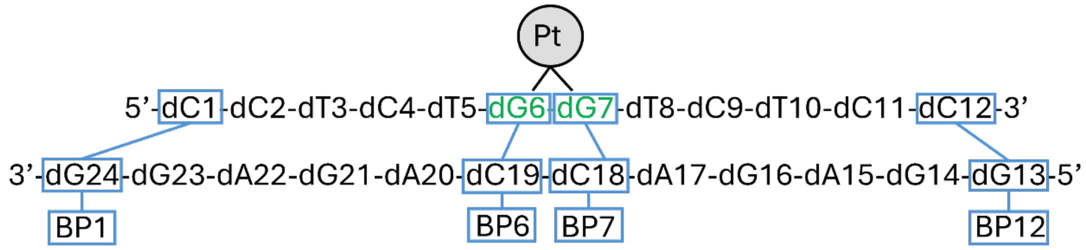

Scheme S1. Residue composition and labelling of the duplex used in the simulations. Each base pair (BP) is labelled after the residue of the 5’-3’ strand, i.e., dC1-dG24 corresponds to BP1.

The cisplatin structure from the original crystallized adduct was either removed, maintained or substituted by one of the heterometallic Pt(II) complexes considered in this work. The cisplatin derivatives were manually inserted in the ds-DNA respecting the original position of the cisplatin Pt atom. The system was parameterized using the OL15 force field[2] for ds-DNA, TIP3P[3] for the water molecules and ions (Mg^{2+} were used to neutralize the system) and a custom force field using the Seminario method[4] as implemented in AmberTools[5] for the platinum ligands. The constant forces of the dihedral angles involving the metal centers were set to 0 kcal·mol^{-1}·rad^{-2} as their strength lies below that of the thermal energy of the medium. According to the Amber20 manual, the implemented Seminario method does not calculate the dihedral bond parameters using a Fourier expansion, but instead uses a harmonic potential. Hence, for the dihedral angles of the heterometallic Pt(II) complexes that do not involve metallic centers the OL15 parameters were used. The frequency calculations were performed resorting to the B3P86/def2-TZVPP protocol, as the performance of this functional to calculate the geometrical parameters for metallic complexes has been reported to be appropriate[6]. In the specific case of the ferrocene, no explicit bonds were defined with the Fe atom but instead 100 kcal·mol^{-1}·Å^{-2} (100 kcal·mol^{-1}·rad^{-2}) harmonic constraints were set for the Fe-C bonds (C-Fe-C angles), similarly to the non-bonded modelling of ferrocene considered in Ref. [7]. This allows the ferrocene to rotate while keeping its key geometrical features. The Lennard-Jones parameters for the metals were extracted from the bibliography[7,8]. Each system was solvated in a truncated octahedron box and periodic boundary conditions were used. The minimum distance between any atom of the system and the edge of the box was set to 12 Å.

The minimization was separated in three different stages to ensure stability of the calculations. First the positions of the hydrogen atoms, then the solvent molecules and finally the whole system were optimized back-to-back. For the three minimizations, 1000 steps were run using a steepest descent algorithm that was then switched to a conjugate gradient algorithm. Then, the system was heated from 100K to 300K using the Langevin thermostat with an integration step of 0.5 fs. The heating was performed in two ramps, setting the increase from 100 K to 273 K in 10000 steps and setting the second increase from 273 K to 300 K in 40000 steps. The initial velocities were fed using a random seed. After heating, a set of five equilibration runs were performed, in which subsequently weaker restraints were applied to the Pt-DNA complex starting from 40 kcal·mol^{-1}·Å^{-2} until no restraints were applied at all. In the last steps, the ensemble was switched from an

NVT to an NPT. The bonds involving hydrogen atoms were restrained using SHAKE[9]. These conditions were set as the standard and three independent 500 ns molecular dynamics trajectories were run for each system, i.e., 1.5 μs for ds-DNA, ds-DNA+**CPT**, ds-DNA+**1**, ds-DNA+**2** and ds-DNA+**3**. All the calculations were performed using PMEMD_CUDA from Amber20[5].

Some properties of the system, as the distortion of the double helix, were measured intercalating equivalent frames of three independent trajectories to ensure convergence of the properties and to avoid drawing conclusions from artifacts happening in specific trajectories. However, the monitoring of other properties as the evolution of the hydrogen bond distances or the non-covalent interactions were specifically analyzed for each trajectory.

The convergence analysis and the geometrical parameters of the dynamics were studied using Cpptraj[5]. The first 100 ns of each trajectory were discarded when analyzing the distortion of the DNA duplex to exclude the stabilization steps. The distortion of the helix was considered using the total helical bend parameter from the program Curves+. As this value might suffer great variations in short timescales, the direct monitoring of its value along the trajectory might lead to confusion. Hence, in this work we decided to use instead the cumulative average value of the helical bend parameter, h_{avg}, and its standard deviation at each iteration of the trajectory. For the comparison of the computational results with the cytotoxic assays, we have defined h_{inv} as follows,

$$h_{inv} = \frac{100 \cdot h_{avg,DNA}}{h_{avg,i}} \quad (1)$$

where $h_{avg,i}$ is the final value of h_{avg} of the *i* species and $h_{avg,DNA}$ is the final h_{avg} value of free ds-DNA. Table S6 presents the calculated values of h_{inv} for each species:

Table S6. Values of h_{inv} for species **1-3**, **CPT** and free ds-DNA.

	ds-DNA+**1**	ds-DNA+**2**	ds-DNA+**3**	ds-DNA+**CPT**	**ds-DNA**
$\boldsymbol{h_{inv}}$	68.18	27.77	11.62	51.72	100

The h_{inv} parameter has no physical meaning, it just facilitates a straightforward comparison with the cell survival ratio from Figures 4, S32-S34 resulting from the cytotoxic assays. As the h_{avg} parameter is directly proportional to the expected cytotoxicity and the cell survival is inversely proportional to the cytotoxicity, we merely considered the inverse of h_{avg} parameter. Other inter- and intra- base pairs parameters accounting for the distortion of the helix such as the tilt, opening, roll and propeller cumulative angles were also monitored.

For the analysis of the non-covalent interactions, the NCIPlot4.0[10,11] code was used. In this type of analysis, the non-covalent interactions within the whole system, i.e, the dodecamer and the corresponding heterobimetallic drug, are calculated along the whole trajectory. The strength and the nature of the non-covalent interactions are characterized based on the magnitude of the reduced density gradient and the sign of the second eigenvalue of the Hessian: the volumes between -0.10 and -0.02 would correspond to strong attractive interactions, from -0.02 to 0.00 to weak attractive interactions, from 0.00 to 0.02 to weak repulsive interactions and from 0.02 to 0.10 to strong repulsive interactions. This analysis was performed using promolecular and SCF (ω97XD/def2-TZVPP) densities.

No restrictions at the terminal base pairs of the dodecamer were applied, except for the ds-DNA+**3** simulations for which intense fraying was observed. Hence, a 240 $kcal \cdot mol^{-1} \cdot Å^{-2}$ harmonic restriction was applied on the distance separating the terminal glycosidic nitrogen atoms for these

specific simulations. Such restriction has previously been applied in the bibliography to this purpose[12]. Comparison of the restricted and unrestricted dynamics of ds-DNA+**3** adduct showed small quantitative differences in the distortion angle h_{avg} along the propagation (Figure S35), and in both cases the distortion fits the experimental trend.

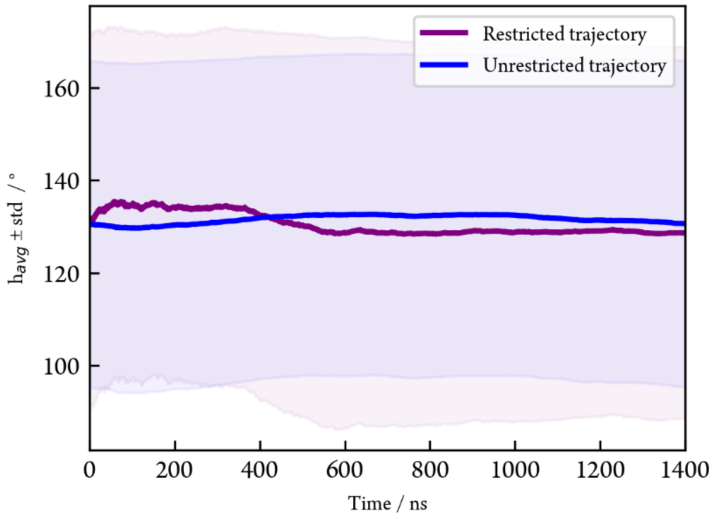

Figure S35. Comparison of the distortion angle h_{avg} between restricted and unrestricted molecular dynamics for ds-DNA+**3.**

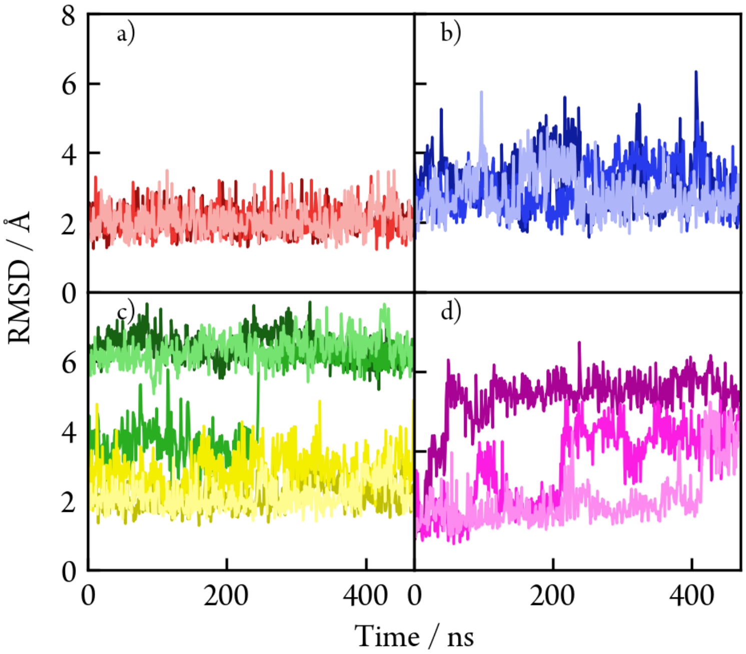

Figure S36. RMSD of the three trajectories (in different tones) run for of a) free ds-DNA, b) ds-DNA+**CPT**, c) ds-DNA+**1** (yellow) and ds-DNA+**2** (green) and d) ds-DNA+**3** (purple).

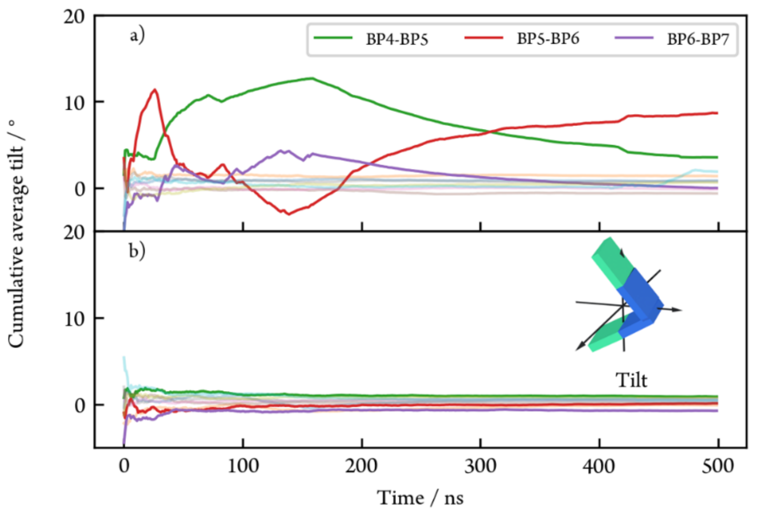

Figure S37. Evolution of the cumulative average tilt angle (inset of panel b) for a selected trajectory of a) ds-DNA+**CPT** and b) free ds-DNA. Each curve denotes the relative tilting of one BP with respect to the next one. The curves corresponding to the tilt angles between consecutive base pairs which are not relevant to the discussion are presented in translucent colours to facilitate the analysis.

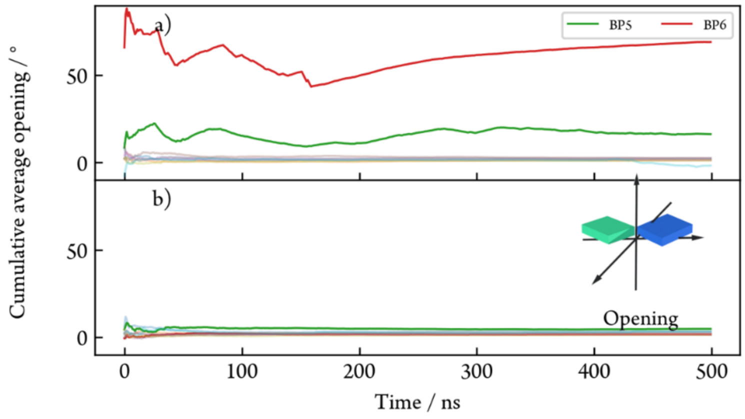

Figure S38. Evolution of the cumulative average opening angle (inset of panel b) for a selected trajectory of a) ds-DNA+**CPT** and b) free ds-DNA. The curves corresponding to the opening angles of the BPs which are not relevant to the discussion are presented in translucent colours to facilitate the analysis.

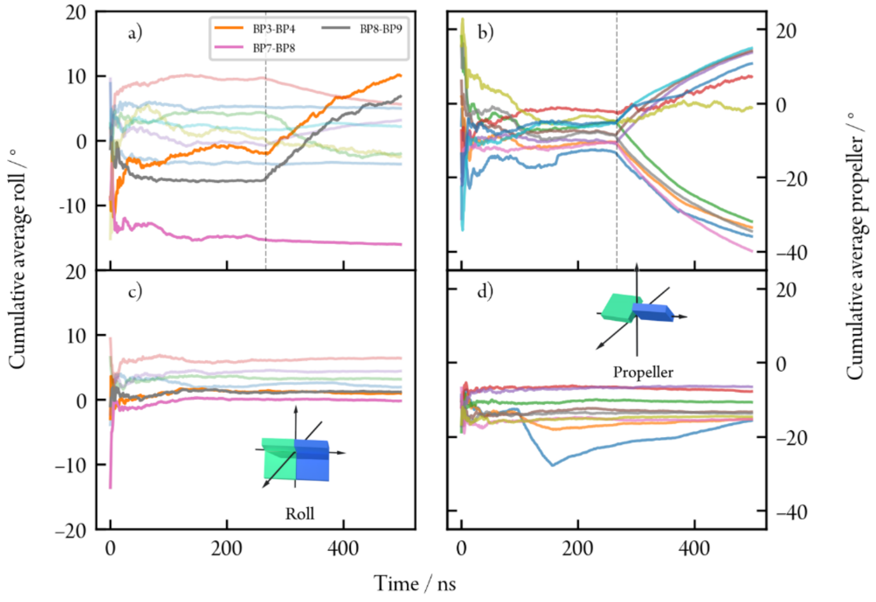

Figure S39. Evolution of the cumulative average roll and propeller angles (insets of panels c and d) for representative trajectories of ds-DNA+**2**, panels a) and b) respectively, and free ds-DNA, panels c) and d) respectively. Each curve denotes the relative roll of one BP compared to the next one and the propeller within a BP. The curves corresponding to the roll and propeller which are not relevant to the discussion are presented in translucent colours to facilitate the analysis.

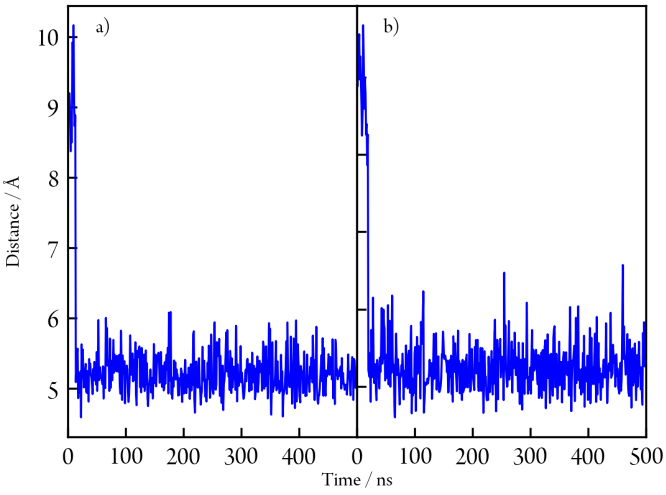

Figure S40. Evolution of the distances (in Angstrom) between the Fe atom and the C6 atom from the dG6 residue along two of the ds-DNA+**2** trajectories.

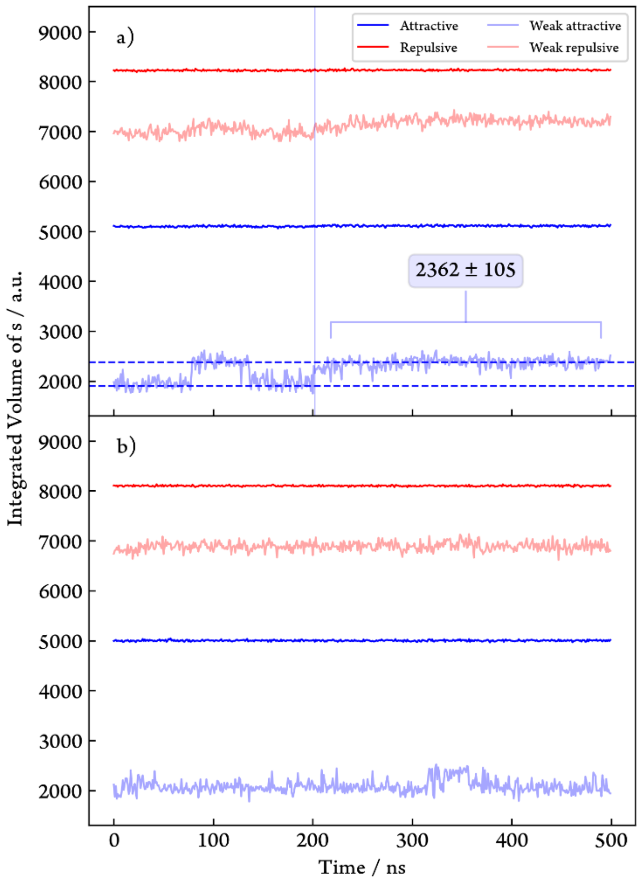

Figure S41. Evolution of the integrated volumes of the reduced density gradient along one of the a) ds-DNA+**2** trajectories and a b) ds-DNA+**1** trajectory decomposed into attractive (blue), weak attractive (light blue), repulsive (red) and weak repulsive (light red) interactions. The blue vertical line in panel a) indicates the moment when the rotation of the ferrocene around the phenyl-cyclopentadienyl bond takes place. The average value and the standard deviation for the total integrated volumes corresponding to the weak attractive interactions after such rotation is also indicated.

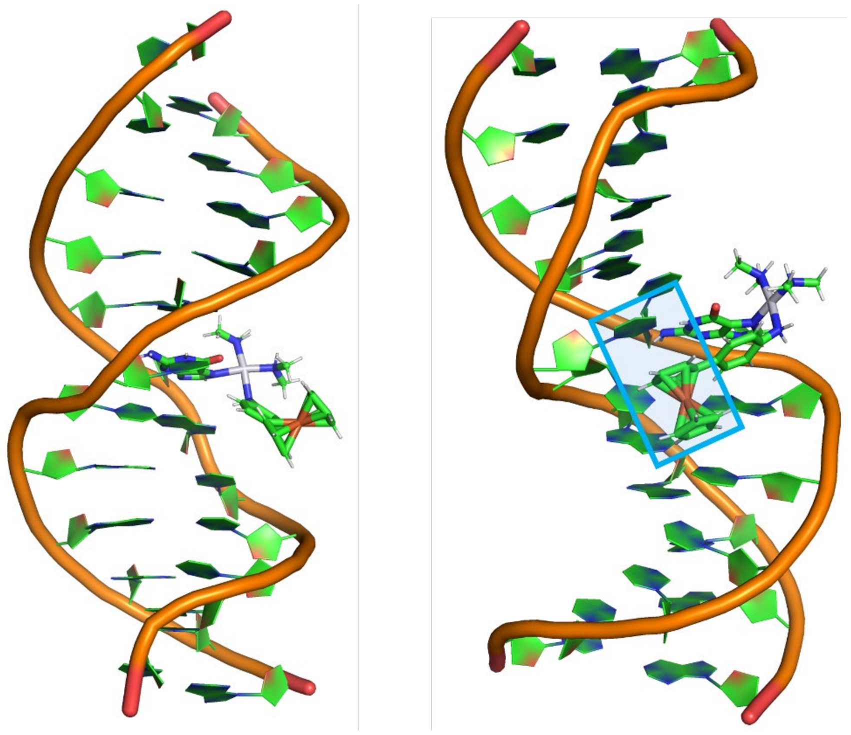

Figure S42. Selected snapshot for the ds-DNA+**2** adduct before (left) and after (right) the rotation of the ferrocene moiety. The blue box highlights the stacking between the ferrocene moiety and nucleotide dC19 contributing to the increase in the total weak attractive interactions in Figures S41 and S43.

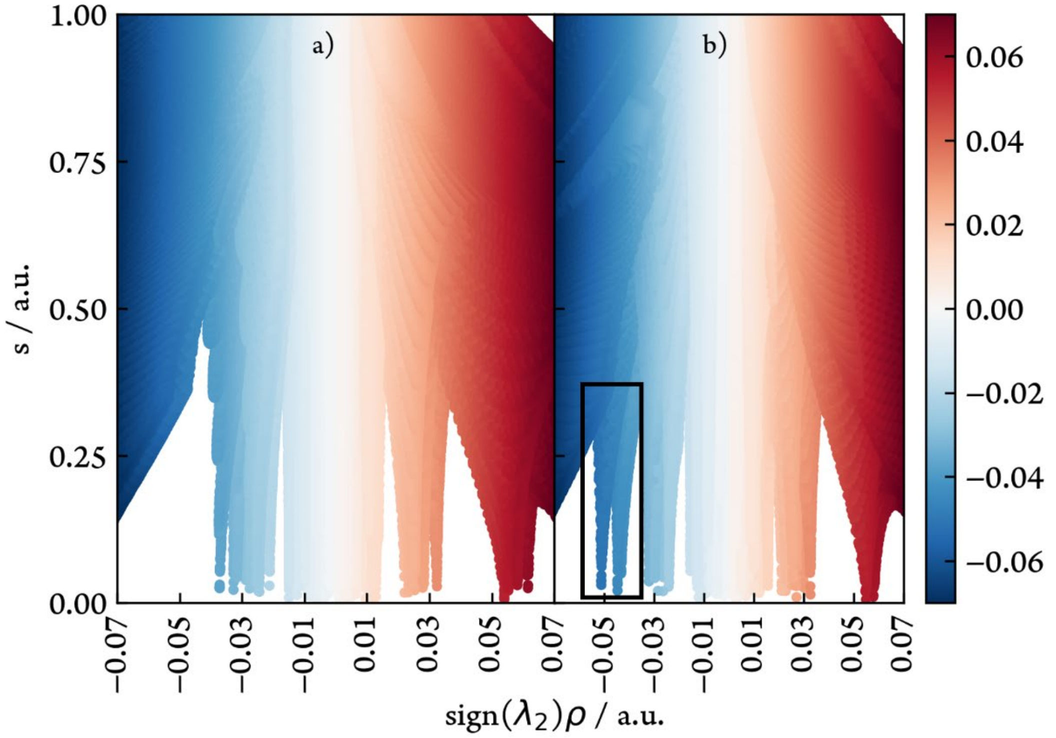

Figure S43. NCI analysis of a cluster consisting of base pairs 6, 7 and 8 and the heteronuclear complex for two representative snapshots of ds-DNA+**2** a) before and b) after the rotation of the ferrocene moiety. The electron densities used for this analysis were obtained from ω97XD/def2-TZVPP single points. The two new strong attractive interactions around -0.05 in panel b) and corresponding to the new hydrogen bonds formed upon rotation of the ferrocene moiety are highlighted with a black box.

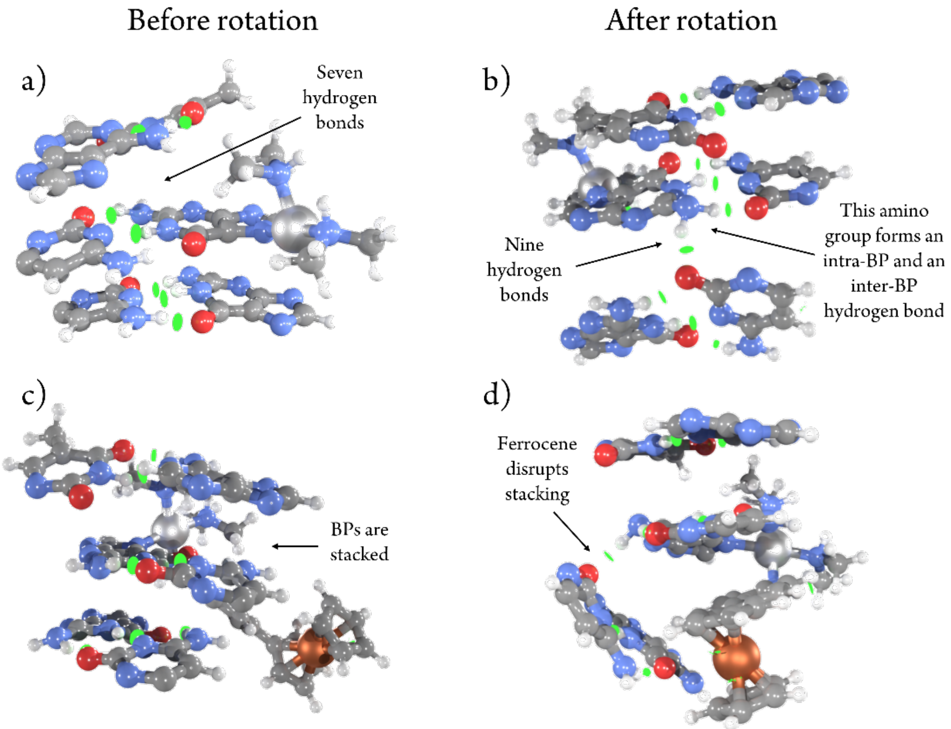

Figure S44. Different views of base pairs 6, 7, 8 and the heteronuclear ligand of two representative snapshots along one of the trajectories of DS-DNA+**2** before (panels a) and c)) and after (panels b) and d)) the rotation of the ferrocene moiety. In panels a) and b) the ferrocene moiety has been omitted for clarity. Green isosurfaces of the reduced density gradient (isovalue=0.30 a.u.) in the regions where $\lambda_2 < -0.20$ is fulfilled are displayed.

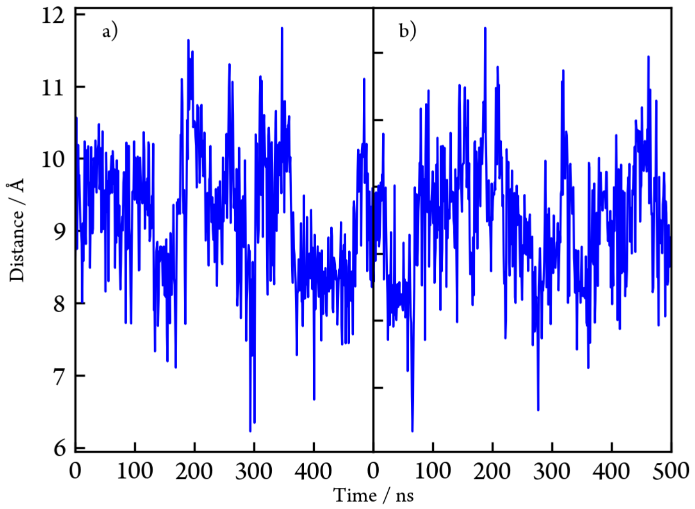

Figure S45. Evolution of the distances (in Angstrom) between the Fe atom from the ligand and the C6 atom from the dG6 residue along two ds-DNA+**1** trajectories.

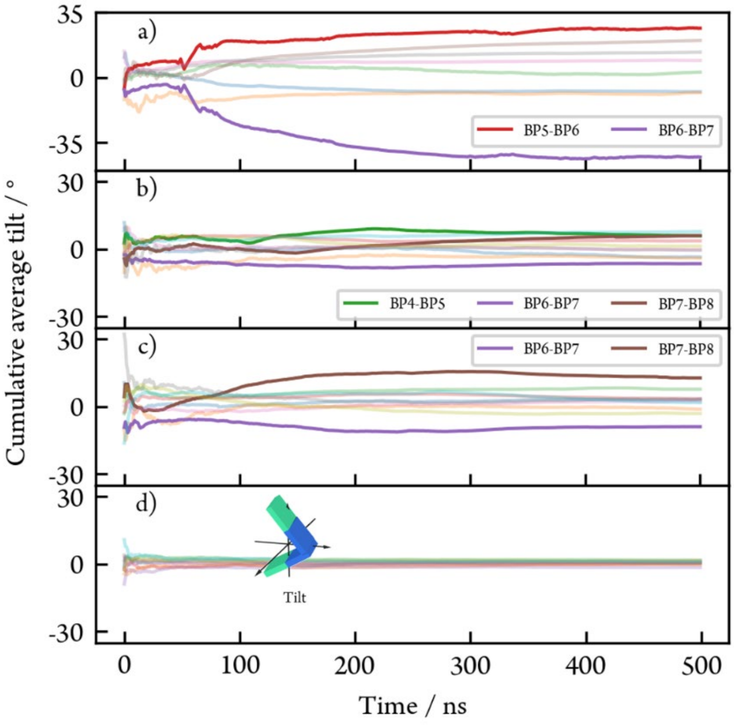

Figure S46. Evolution of the cumulative average tilt angle for all trajectories of ds-DNA+**3** (panels a, b and c) and free ds-DNA (panel d). Each curve denotes the tilting of one BP with respect to the next one. The curves corresponding to the tilt angles between consecutive base pairs which are not relevant to the discussion are presented in translucent colours to facilitate the analysis.

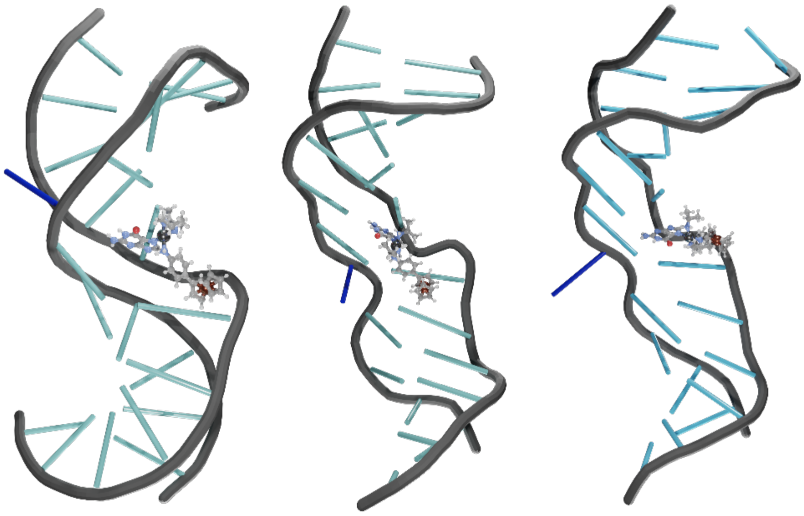

Figure S47. Representative snapshots for each trajectory propagated of the ds-DNA+**3** adduct. The nucleotide that has been pushed out of the nucleobase pairing cavity due to the distortion of the double helix has been highlighted in dark blue.

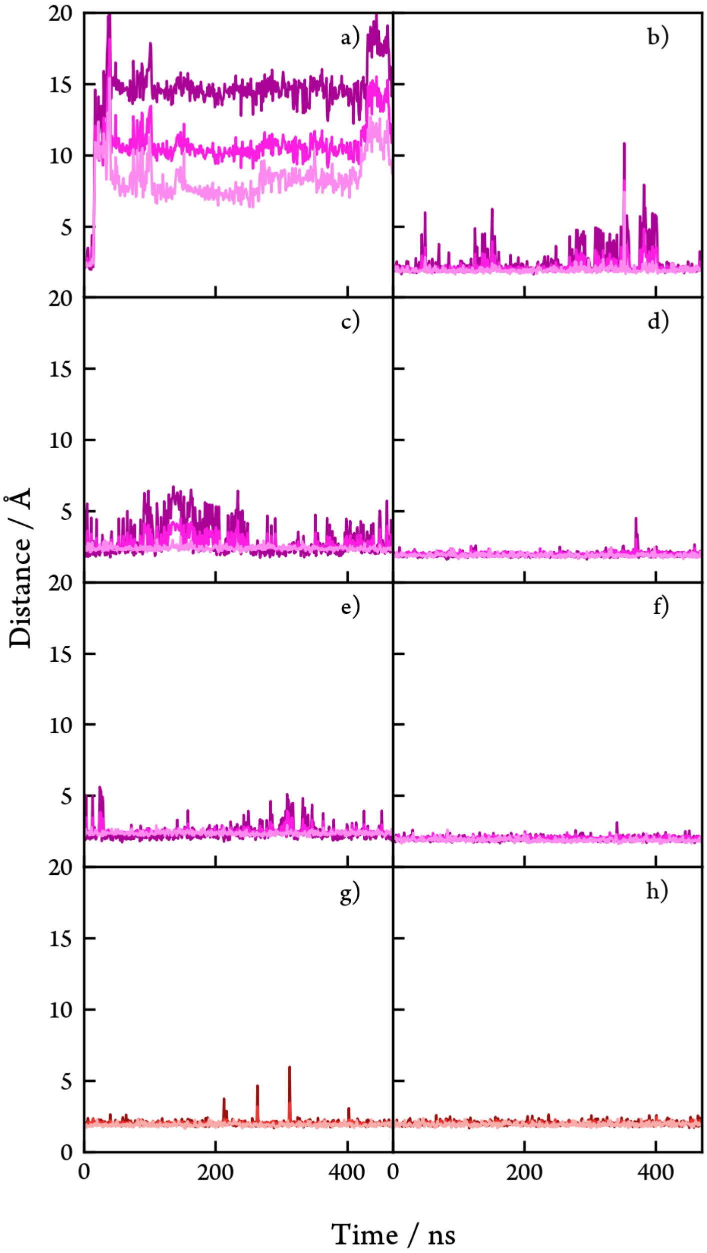

Figure S48. Evolution of the three hydrogen bond distances shown in different tones of the dG6-dC19 (panels, a, c, and e) and dG7-dC18 (panels, b, d and f) base pairs for the three trajectories (first, second and third rows respectively) of ds-DNA+**3**. Panels g) and h) present the same hydrogen bond distances for the ds-DNA trajectories, to be used as reference.

8. REFERENCES

(1) Todd, R. C.; Lippard, S. J. Structure of Duplex DNA Containing the Cisplatin 1,2-{$Pt(NH_3)_2$}$^{2+}$-d(GpG) Cross-Link at 1.77Å Resolution. *J. Inorg. Biochem.* **2010**, *104* (9), 902–908.

(2) Galindo-Murillo, R.; Robertson, J. C.; Zgarbová, M.; Šponer, J.; Otyepka, M.; Jurečka, P.; Cheatham, T. E. Assessing the Current State of Amber Force Field Modifications for DNA. *J. Chem. Theory Comput.* **2016**, *12* (8), 4114–4127.

(3) Jorgensen, W. L.; Chandrasekhar, J.; Madura, J. D.; Impey, R. W.; Klein, M. L. Comparison of Simple Potential Functions for Simulating Liquid Water. *J. Chem. Phys.* **1983**, *79* (2), 926–935.

(4) Seminario, J. M. Calculation of Intramolecular Force Fields from Second-Derivative Tensors. *Int. J. Quantum Chem.* **1996**, *60* (7), 1271–1277.

(5) Case, D. A.; Belfon, K.; Ben-Shalom, I.Y.; Brozell, S.R.; Cerutti, D.S.; Cheatham, T.E.; Cruzeiro, V.W.D.; Darden, T.A.; Duke, R.E.; Giambasu, G.; Gilson, M.K.; Gohlke, H.; Goetz, A.W.; Harris, R.; Izadi, S.; Izmailov, S.A.; Kasavajhala, K.; Kovalenko, A.; Krasny, R.; Kurtzman, T.; Lee, T.S.; LeGrand, S.; Li, P.; Lin, C.; Liu, J.; Luchko, T.; Luo, R.; Man, V.; Merz, K.M.; Miao, Y.; Mikhailovskii, O.; Monard, G.; Nguyen, H.; Onufriev, A.; Pan, F.; Pantano, S.; Qi, R.; Roe, D.R.; Roitberg, A.; Sagui, C.; Schott-Verdugo, S.; Shen, J.; Simmerling, C.L.; Skrynnikov, N.R.; Smith, J.; Swails, J.; Walker, R.C.; Wang, J.; Wilson, L.; Wolf, R.M.; Wu, X.; Xiong, Y.; Xue, Y.; York, D.M.; Kollman, P.A. Amber20. *Amber* **2020**, *San Francisco* (University of California).

(6) Bühl, M.; Reimann, C.; Pantazis, D. A.; Bredow, T.; Neese, F. Geometries of Third-Row Transition-Metal Complexes from Density-Functional Theory. *J. Chem. Theory Comput.* **2008**, *4* (9), 1449–1459.

(7) De Hatten, X.; Cournia, Z.; Huc, I.; Smith, J. C.; Metzler-Nolte, N. Force-Field Development and Molecular Dynamics Simulations of Ferrocene-Peptide Conjugates as a Scaffold for Hydrogenase Mimics. *Chemistry - A European Journal* **2007**, *13* (29), 8139–8152.

(8) Ruano, L.; Cárdenas, G.; Nogueira, J. J. The Permeation Mechanism of Cisplatin Through a Dioleoylphosphocholine Bilayer. *ChemPhysChem* **2021**, *22* (12), 1251–1261.

(9) Miyamoto, S.; Kollman, P. A. SETTLE: An Analytical Version of the SHAKE and RATTLE Algorithm for Rigid Water Models. *J. Comput. Chem.* **1992**, *13* (8), 952–962.

(10) Laplaza, R.; Peccati, F.; A. Boto, R.; Quan, C.; Carbone, A.; Piquemal, J. P.; Maday, Y.; Contreras-García, J. NCIPLOT and the Analysis of Noncovalent Interactions Using the Reduced Density Gradient. *Wiley Interdisciplinary Reviews: Computational Molecular Science* **2021**, *11* (2), e1497.

(11) Johnson, E. R.; Keinan, S.; Mori-Sánchez, P.; Contreras-García, J.; Cohen, A. J.; Yang, W. Revealing Noncovalent Interactions. *J. Am. Chem. Soc.* **2010**, *132* (18), 6498–6506.

(12) Armeev, G. A.; Kniazeva, A. S.; Komarova, G. A.; Kirpichnikov, M. P.; Shaytan, A. K. Histone Dynamics Mediate DNA Unwrapping and Sliding in Nucleosomes. *Nat. Commun.* **2021**, *12* (1), 2387.
